# A Scoping Review of Methodologies Exploring Diet and Health Outcomes in Lactating Women: What Has Been Done and Where to Next?

**DOI:** 10.1093/nutrit/nuae228

**Published:** 2025-02-17

**Authors:** Sofa Rahmannia, Gina Arena, Kevin Murray, Ana D Sakinah, Yughni A Thariqi, Aly Diana, Siobhan Hickling

**Affiliations:** School of Population and Global Health, The University of Western Australia, Crawley, WA 6009, Australia; Faculty of Medicine, Universitas Pasundan, Bandung, West Java 40117, Indonesia; Medical School, The University of Western Australia, Nedlands, WA 6009, Australia; Telethon Kids Institute, Nedlands, WA 6009, Australia; School of Population and Global Health, The University of Western Australia, Crawley, WA 6009, Australia; Nutrition Working Group, Faculty of Medicine, Universitas Padjadjaran, Bandung, West Java 45363, Indonesia; Nutrition Working Group, Faculty of Medicine, Universitas Padjadjaran, Bandung, West Java 45363, Indonesia; Nutrition Working Group, Faculty of Medicine, Universitas Padjadjaran, Bandung, West Java 45363, Indonesia; Department of Public Health, Faculty of Medicine, Universitas Padjadjaran, Bandung, West Java 45363, Indonesia; School of Population and Global Health, The University of Western Australia, Crawley, WA 6009, Australia

**Keywords:** dietary variables, breastfeeding outcome, lactating women, dietary guidelines, nutritional epidemiology

## Abstract

Developing dietary guidelines for lactating women presents significant challenges, due to limited evidence being available on their specific nutrient needs and the biological impacts of various dietary dimensions. Current dietary recommendations often rely on data from nonlactating women, leading to potential inaccuracies. The relationship between diet and health outcomes in lactating women remains underexplored, particularly across different dietary dimensions, such as nutrients, food groups, dietary patterns, and other specific dietary variables. The aims of this scoping review were to map the diverse methodologies employed in research into maternal diet during lactation, to identify the current gaps, and to suggest areas for future investigation. The review focused on the dietary variables studied in relation to breastfeeding outcomes, and offers insights into the current state of lactation nutrition research. A comprehensive search was conducted in the CINAHL, MEDLINE, and Embase databases up to December 13, 2022. The included studies encompassed original quantitative research on dietary intake among lactating women and any associated outcomes. Data extracted included study characteristics, dietary variables, and outcome measures, and they were analyzed using descriptive statistics and pivot tables. Of the 1666 relevant studies identified, 231 met the inclusion criteria. Most research was conducted in high-income and upper-middle-income countries. Studies mainly focused on relatively short lactation durations, nutrient-based dietary dimensions, and maternal outcomes, particularly breast milk composition. Maternal metabolic status and child outcomes, such as infant micronutrient status and longitudinal growth, were underexplored. Specific dietary variables included meal frequency and nutrient intake from various food sources. Commonly adjusted covariates were maternal age and socio-economic status, while contraceptive use and sanitation were often overlooked. Many areas of research concerning the diet–health relationship in lactating women remain unexplored. Filling these gaps will gather evidence to inform the development of dietary guidelines for this population.

## INTRODUCTION

Developing dietary guidelines for lactating women requires robust evidence specific to this population that accurately indicates daily nutrient needs. There is a substantial gap in the evidence available regarding the biological effects of individual nutrients, the understanding of which is essential for estimating nutrient requirements using clinical approach.^1^ Given these limitation, the factorial approach remains the primary method for determining nutrient requirements.^1–3^ This approach estimates nutrient requirements based on the assumption that nutrient input should equal nutrient loss. Data obtained using this approach include rates of absorption, utilization, storage, and excretion of nutrients, in relation to intake. However, the current nutrient requirements for lactating women are typically determined by extrapolation from data on nonlactating women, with breast milk secretion being additionally taken into account in the determination of nutrient losses.^1^^,^^4–8^

It is well established that nutrient absorption rates in lactating women are higher than in nonpregnant, nonlactating women, likely due to a homeostatic mechanism that ensures sufficient nutrients for breast milk production.^1–3^ However, earlier studies have indicated that this homeostatic process in lactating women also affects excretion rates, particularly through increased intestinal retention. This process helps maintain the relatively constant nutrient composition of breast milk, even among women with low dietary intake.^9^ Despite these findings, nutrient requirement estimations still rely on nutrient loss data from nonlactating women. Consequently, the resulting estimated intake requirements for many nutrients may be higher than is necessary. More research is needed on lactating women to improve understanding of how to estimate their specific nutrient losses and the homeostatic process, and thereby determine their nutrient needs.

Furthermore, the data on breast milk micronutrient composition used by international agencies to define nutrient requirements have largely been obtained from studies with small sample sizes, typically fewer than 10 lactating women. These studies have indicated a wide range of micronutrient concentrations, a lack of longitudinal data, and the use of heterogeneous methods of breast milk collection.^10^^,^^11^ This further highlights the need for more robust research to inform dietary recommendations during lactation.

Food-Based Dietary Guidelines (FBDGs) are designed to communicate the nutrient requirement standards in user-friendly terms by suggesting food patterns that fulfil these requirements.^12^^,^^13^ Evidence for the health benefits of food consumption is frequently utilized to establish minimum/maximum parameters for modeling daily servings of specific foods.^13^ However, each food group has unique characteristics, especially regarding nutrient bioavailability profiles.^14^ These characteristics have not yet been used to determine the ideal combinations of food groups, or been reflected in FBDGs. Instead, the current factors affecting the FBDGs relate to affordability, availability, and local dietary habits.^15–18^ To date, the health benefits of key food groups have not been extensively studied in lactating populations. Further research is needed on the effects of differences in food consumption on health outcomes during lactation if we are to optimize the dietary guidelines and improve health outcomes for both mothers and their children.

Recent changes in the approaches to developing dietary guidelines, demonstrated by the development of the Dietary Guidelines for Americans (DGA) 2020–2025, highlight the importance of considering overall dietary patterns, rather than focusing solely on individual nutrients or foods. The DGA defines dietary patterns as “the quantities, proportions, variety, or combination of different foods, drinks, and nutrients (when available) in diets, and the frequency with which they are habitually consumed.”^19^ However, defining these patterns poses challenges, due to variations in the components of the food groups being consumed. This has made it difficult in some studies to draw definitive conclusions about what constitutes a healthy diet. Despite systematic efforts to identify relevant studies on dietary patterns and their impacts on lactation outcomes for both mothers and children, many research questions regarding lactation outcomes remain unanswered, due to either no evidence or limited evidence being available.^20–24^ This indicates that there is still much ground to cover in research within this population if we are to generate comprehensive dietary recommendations.

Interest in lactation nutrition research has trailed behind interest in pregnancy nutrition research. Most studies have focused on describing dietary adequacy or nutritional status, rather than comprehensively exploring the relationships between maternal diet, maternal nutrient status, breast milk composition, and infant nutritional status. This scoping review aimed, therefore, to bridge this gap by mapping the dietary variables studied in relation to breastfeeding outcomes. By identifying the various nutrients, food groups, dietary patterns, and other specific dietary variables investigated thus far, as well as the methodologies and geographical distributions of the research, this review sought to provide insights into the current state of lactation nutrition research. The actual results of the analyses conducted are not discussed here, as each dietary variable and outcome requires a separate systematic review for the drawing of definitive conclusions. Nonetheless, this review highlights areas that require further investigation, assisting the prioritizing of future research, with the goal of improving dietary recommendations and health outcomes for both mothers and their children.

## METHOD

### Identification of Research Questions

The primary objective of this scoping review was to consolidate the existing dietary variables and identify research gaps in the analysis of diet and health outcomes during lactation. Specifically, this review has clarified which aspects of lactating women’s diets—including nutrients, food groups, dietary patterns, and other specific dietary factors—have undergone extensive analysis, and which have remained underexplored.

In addition to the primary aims, secondary questions have been addressed relating to the outcomes, geographical representation, and methodological approaches employed in the examination of the relationship between diet and health outcomes in lactating women:

1. What specific health outcomes for both lactating women and their children have been comprehensively studied in relation to diet, and which areas remain inadequately investigated or poorly understood?
2. Are there geographical disparities in the availability of research data?
3. What methodologies have been predominantly utilized to assess dietary intake in studies involving lactating women?
4. What inclusion and exclusion criteria have commonly been employed in designing studies on lactating women’s diets?
5. Are particular stages or durations of lactation receiving more attention in research on diet and health outcomes, and are there gaps in our understanding of the long-term effects of maternal diet during lactation?
6. How have researchers adjusted for confounding variables when examining the relationship between diet and health outcomes during lactation?

### Search Strategy

A comprehensive search strategy was devised to identify relevant studies, targeting the key databases CINAHL, MEDLINE, and Embase. The search was conducted covering the period from the inception of each database up to December 13, 2022. The search strategy was constructed using a combination of Medical Subject Headings (MeSH) terms and keywords related to dietary assessment, diet, and lactation.

The search strategy for MEDLINE was structured as follows:

1. Search for articles related to diet surveys or diet records using MeSH terms: “exp Diet Surveys/” or “exp diet records/”.
2. Identify articles containing terms related to diet, such as diet*, food*, or weigh*, in conjunction with terms related to data collection methods, including record*, recall*, questionnaire*, diary, or diaries, using the adjacency operator (adj2): ([diet* or food* or weigh*] adj2 [record* or recall* or questionnaire* or diary or diaries]).ti, ab.
3. Combine the results of steps 1 and 2 using the Boolean operator “OR”: 1 or 2.
4. Search for articles related to lactation, postpartum period, or breastfeeding using MeSH terms: “exp Lactation/” or “exp Postpartum period/” or “exp Breast Feeding/”.
5. Identify articles containing terms related to lactation or breastfeeding, such as lactating, lactation, breastfeeding, postpartum, or post-partum, in conjunction with terms related to women or mothers using the adjacency operator (adj2): ([lactating or lactation or breastfeeding or postpartum or post-partum] adj2 [women or woman or mother* or female]).ti, ab.
6. Combine the results of steps 4 and 5 using the Boolean operator “OR”: 4 or 5.
7. Combine the results of steps 3 and 6 using the Boolean operator “AND”: 3 and 6.

No keywords related to outcomes were used, to avoid limiting the discovery of articles based on specific outcomes. The same search strategy was also applied to other databases. Articles examining outcomes were distinguished from those that did not during the screening phase.

The inclusion criteria for this scoping review specified original quantitative research, including observational studies and clinical trials, focusing on measuring dietary intake among lactating women and analyzing dietary data with any outcome variable. Excluded studies involved experimental research on animals or in vitro models, and observational studies measuring exposure to intoxicants such as mercury. Limits were imposed to exclusively include original articles published in peer-reviewed journals and written in English.

### Study Selection Process and Data Extraction

The study selection process was conducted in Covidence^25^ by reviewers possessing nutrition or health science expertise. Two independent reviewers screened titles, abstracts, and full-text articles based on pre-defined inclusion and exclusion criteria. Discrepancies were resolved through discussion. The extracted data included the characteristics of the studies, as well as the diet and outcome variables being analyzed. Study characteristics included details such as author(s), year of publication, country, population, inclusion and exclusion criteria, number of participants, dietary assessment method, number of measurement days, and study design.

### Synthesis of Results

The data extracted from Covidence was exported into Excel for further analysis. Descriptive statistics were used to summarize the characteristics of the studies included in this scoping review, with most variables being presented as percentages (except for number of participants, which was presented as median and range). Certain studies had multiple descriptors for some of the characteristics, such as the country of study, or the type of dietary assessment used. Thus, the numbers of studies with these characteristics did not necessarily match the total number of studies included in the review.

**Figure 1.**
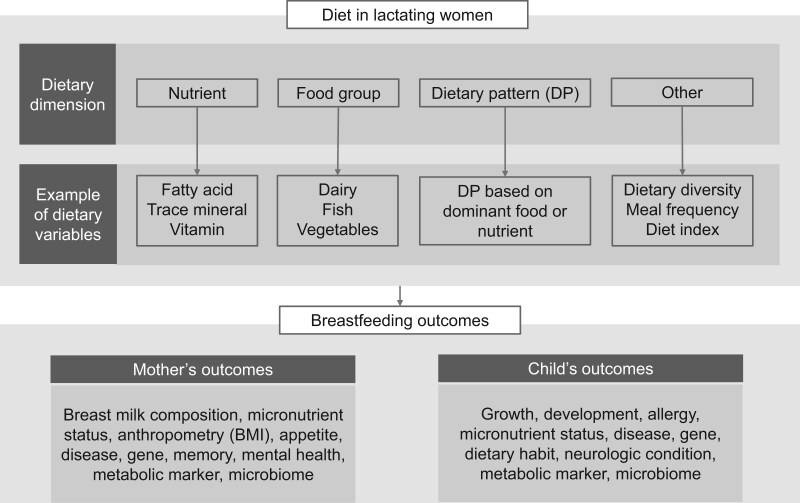
Overview of Diet–Outcome Research in Lactating Women

The dietary variable (D) and outcome variable (O) information from each article extracted in Covidence followed a D × O matrix format. The extracted data resulted in a wide-shape dataset (eg, D1O1, D1O2, D1O3). Subsequently, this dataset was converted into a long-shape format, outlining an analysis of each dietary variable with each outcome variable. This format comprehensively represented the total number of analyses conducted across all articles. Similar dietary and outcome variables were renamed in a long-shape format database to ensure consistency. Dietary variables were coded based on their type, such as nutrient, food, dietary pattern, or other, as detailed in Tables S1–S4. Similarly, outcome variables were categorized into outcomes for mothers or outcomes for children, and included micronutrient status, growth, development, and disease, as outlined in Table S5. An overview of the diet–outcome variables in research on lactating women is shown in Figure 1.

The long-shape dataset was analyzed using Excel’s pivot table feature to generate cross-tabulations of the numbers of articles based on each dietary variable and outcome. Due to the large number of dietary and outcome variables, it was not feasible to present the exact details in tabular form. However, the wide- and long-shape datasets are accessible for reference. These cross-tabulations provide insights into the distribution of the research and identify areas of underexplored analysis in the maternal diet in relation to outcomes for both mothers and children.

Additionally, adjustment variables used in the analyses were summarized based on their usage frequency in relation to the outcomes under investigation. This presentation provide an overview of the distribution of the adjustment variables in relation to the studied outcomes.

**Table 1.** Characteristics of the Studies Included in the Scoping Review (*n* = 231)

Characteristics	I	%
**Country income** ^a^		
Low income	3	1%
Lower middle income	20	8%
Upper middle income	74	31%
High income	146	60%
**Region** ^a^		
Africa	10	4%
Asia and the Pacific	91	37%
Europe	69	28%
Latin America and the Caribbean	15	6%
Near East	8	3%
North America	50	21%
**Population studied**		
Lactating women only	148	64%
Pregnant and lactating women	32	14%
Lactating women and infants/children	41	18%
Women, including lactating women	6	3%
General population, including lactating women	4	2%
**Period of lactation**		
Less than 1 month	9	4%
Less than 6 months	146	63%
Less than 1 year	39	17%
Less than 2 years	33	14%
More than 2 years	4	2%
**Inclusion criteria applied in studies** ^a^		
Breastfeeding only 1 child	59	26%
Birth weight >2500 g	29	13%
Delivered full-term child	55	24%
Currently exclusive breastfeeding	41	18%
Currently predominant breastfeeding	6	3%
Currently partial breastfeeding	6	3%
Adult (over 18 years old)	46	20%
Other	121	52%
**Exclusion criteria applied in studies** ^a^		
Reported implausible energy intake	8	3%
Taking vitamin/mineral supplement	15	6%
Known medical problems/chronic disease	126	55%
Infants born with congenital disabilities	30	13%
Smoker	48	21%
Drinker	24	10%
Other	79	34%
**Health status**		
Healthy participants	218	95%
Participants with specific medical conditions	12	5%
**Median number of participants (range)**	100 (10–9601)	
**Diet assessment method** ^a^		
Food Frequency Questionnaire (FFQ)	85	37%
Semi-quantitative FFQ	18	8%
Food recall	74	32%
Food weight record	12	5%
Food diary	11	5%
Diet record (unspecified)	56	24%
Other	28	12%
**Number of days of diet measurement**		
1	71	37%
2	24	13%
3	70	36%
>3	27	14%
**Study design**		
Cross-sectional	147	64%
Case–control	7	3%
Cohort	62	27%
Clinical trial	12	5%
Other: longitudinal study	3	1%

a Multiple characteristics can be present within 1 study.

**Table 2.** General Distribution of Research Articles (*n* = 231) Based on Diet Dimensions and Mother/Child Breastfeeding Outcomes

**Diet dimension** ^a^	**Mother’s outcomes** ^b^	**Child’s outcomes** ^b^
	*n* (%)	*n* (%)
Nutrient	144 (62%)	14 (6%)
Food group	55 (24%)	14 (6%)
Dietary pattern	13 (6%)	2 (1%)
Other	61 (26%)	16 (7%)

a Detailed information on each dietary dimension can be found in supplementary tables 1–4.

b Some research articles have more than 1 dietary dimension.

**Table 3.** Research Article Distribution Among Lactating Women’s Dietary Variables and Mother Outcomes

Dietary variables	Mother’s outcomes										
	Numbers of research articles (proportion from each dimension: Nutrient, food group, dietary pattern, and others)										
	Micronutrient status	Breast milk	Anthropometry	Diet habit	Disease	Gene	Memory	Mental health	Metabolic	Microbiome	Total
**Nutrient**	**30**	**91**	**14**	**1**	**1**	**0**	**1**	**2**	**1**	**3**	**144**
Alkaloid		2 (2%)	1 (7%)					1 (50%)			4 (3%)
Amino acid	1 (3%)	4 (4%)									5 (3%)
Antinutrient	1 (3%)										1 (1%)
Carb derivate										1 (33%)	1 (1%)
Electrolyte	1 (3%)	4 (4%)									5 (3%)
Energy	2 (7%)	21 (23%)	10 (71%)	1 (100%)	1 (100%)			1 (50%)		2 (67%)	38 (26%)
Fatty acid	1 (3%)	24 (26%)	1 (7%)						1 (100%)	2 (67%)	29 (20%)
Fiber		3 (3%)	3 (21%)						1 (100%)	1 (33%)	8 (6%)
Flavonoid		3 (3%)					1 (100%)				4 (3%)
Macronutrient	4 (13%)	34 (37%)	6 (43%)					1 (50%)		2 (67%)	47 (33%)
Mineral	10 (33%)	10 (11%)	1 (7%)								21 (15%)
Mixed nutrient		4 (4%)	0%							1 (33%)	5 (3%)
Phenolic compound							1 (100%)				1 (1%)
Phytonutrient							1 (100%)				1 (1%)
Polyphenol		3 (3%)					1 (100%)				4 (3%)
Trace mineral	9 (30%)	13 (14%)						1 (50%)		1 (33%)	24 (17%)
Vitamin A	3 (10%)	19 (21%)			1 (100%)						23 (16%)
Vitamin B	2 (7%)	9 (10%)	1 (7%)		1 (100%)			2 (100%)		1 (33%)	16 (11%)
Vitamin C	2 (7%)	3 (3%)									5 (3%)
Vitamin D	4 (13%)	3 (3%)						1 (50%)			8 (6%)
Vitamin E		8 (9%)									8 (6%)
**Food groups**	**6**	**35**	**5**	**0**	**3**	**2**	**0**	**3**	**0**	**1**	**55**
Bean and nut	2 (33%)	11 (31%)			1 (33%)						14 (25%)
Dairy	2 (33%)	15 (43%)	1 (20%)		1 (33%)	1 (50%)					20 (36%)
Drink		3 (9%)	2 (40%)							1 (100%)	6 (11%)
Egg	3 (50%)	9 (26%)	1 (20%)		1 (33%)						14 (25%)
Fish	4 (67%)	16 (46%)	2 (40%)		2 (67%)	1 (50%)					25 (45%)
Fruit		13 (37%)	2 (40%)		1 (33%)			1 (33%)		1 (100%)	18 (33%)
Grain	1 (17%)	11 (31%)	1 (20%)			1 (50%)		1 (33%)		1 (100%)	16 (29%)
Herb		1 (3%)			2 (67%)						3 (5%)
Meat	2 (33%)	15 (43%)	1 (20%)		1 (33%)	1 (50%)					20 (36%)
Mixed food		3 (9%)						1 (33%)			4 (7%)
Oil	1 (17%)	6 (17%)	1 (20%)								8 (15%)
Snack		1 (3%)				1 (50%)					2 (4%)
Specific food	2 (33%)	3 (9%)	1 (20%)							1 (100%)	7 (13%)
Vegetable	2 (33%)	14 (40%)	1 (20%)		2 (67%)			2 (67%)			21 (38%)
**Dietary pattern**	**1**	**7**	**4**	**0**	**0**	**0**	**0**	**1**	**0**	**0**	**13**
Dominant food		1 (14%)	1 (25%)					1 (100%)			3 (23%)
Dominant nutrient		2 (29%)	1 (25%)								3 (23%)
Predefined	1 (100%)	5 (71%)	2 (50%)								8 (62%)
**Others**	**13**	**19**	**17**	**1**	**2**	**1**	**0**	**4**	**2**	**2**	**61**
Feature of food	1 (8%)		2 (12%)							1 (50%)	4 (7%)
Habit		1 (5%)	1 (6%)	1 (100%)				1 (25%)			4 (7%)
Index	1 (8%)		5 (29%)					2 (50%)	2 (100%)		10 (16%)
Mixed food		1 (5%)									1 (2%)
Nutrient food source	1 (8%)	5 (26%)			1 (50%)					1 (50%)	8 (13%)
Other substance											0 (0%)
Sugar		3 (16%)	3 (18%)			1 (100%)		1 (25%)			8 (13%)
Supplement	4 (31%)	3 (16%)									7 (11%)
Timing	1 (8%)	1 (5%)	4 (24%)								6 (10%)
Type of food	3 (23%)	5 (26%)	4 (24%)		1 (50%)					1 (50%)	14 (23%)
Variety of food	3 (23%)	4 (21%)	4 (24%)					1 (25%)			12 (20%)

The percentage is calculated based on the proportion of articles investigating each dietary variable relative to the total number of articles within the same dietary dimension (nutrient, food group, dietary pattern, and other dietary dimensions).

**Table 4.** Research Article Distribution Among Lactating Women’s Dietary Variables and Child Outcomes

Dietary variables	Child’s outcomes										
	Numbers of research articles (proportion from each dimension: Nutrient, food group, dietary pattern, and others)										
	Micronutrient status	Development	Growth	Allergy	Disease	Gene	Infant diet	Metabolic	Microbiome	Neurologic	Total
**Nutrient**	**1**	**1**	**4**	**3**	**1**	**1**	**0**	**1**	**1**	**1**	**14**
Alkaloid	0%										0 (0%)
Amino acid											0 (0%)
Antinutrient											0 (0%)
Carb derivate		1 (100%)									1 (7%)
Electrolyte											0 (0%)
Energy		1 (100%)	2 (50%)							1 (100%)	4 (29%)
Fatty acid	1 (100%)		1 (25%)	2 (67%)	1 (100%)					1 (100%)	6 (43%)
Fiber			1 (25%)						1 (100%)	1 (100%)	3 (21%)
Flavonoid											0 (0%)
Macronutrient			3 (75%)					1 (100%)	1 (100%)	1 (100%)	6 (43%)
Mineral											0 (0%)
Mixed nutrient											0 (0%)
Phenolic compound											0 (0%)
Phytonutrient											0 (0%)
Polyphenol											0 (0%)
Trace mineral											0 (0%)
Vitamin A										1 (100%)	1 (7%)
Vitamin B				1 (33%)		1 (100%)				1 (100%)	3 (21%)
Vitamin C											0 (0%)
Vitamin D											0 (0%)
Vitamin E											0 (0%)
**Food groups**	**1**	**2**	**3**	**3**	**2**	**0**	**2**	**1**	**0**	**0**	**14**
Bean and nut											0 (0%)
Dairy			2 (67%)	2 (67%)				1 (100%)			5 (36%)
Drink											0 (0%)
Egg											0 (0%)
Fish		1 (50%)		2 (67%)							3 (21%)
Fruit		1 (50%)	1 (33%)	2 (67%)			2 (100%)	1 (100%)			7 (50%)
Grain			1 (33%)	1 (33%)				1 (100%)			3 (21%)
Herb											0 (0%)
Meat	1 (100%)			1 (33%)	2 (100%)						4 (29%)
Mixed food				1 (33%)							1 (7%)
Oil	1 (100%)		1 (33%)	1 (33%)	1 (50%)			1 (100%)			5 (36%)
Snack							1 (50%)				1 (7%)
Specific food			1 (33%)								1 (7%)
Vegetable				1 (33%)			2 (100%)				3 (21%)
**Dietary pattern**	**0**	**0**	**0**	**1**	**0**	**0**	**1**	**0**	**0**	**0**	**2**
Dominant food							1 (100%)				1 (50%)
Dominant nutrient				1 (100%)			1 (100%)				2 (100%)
Predefined											0 (0%)
**Others**	**0**	**2**	**8**	**2**	**1**	**0**	**2**	**0**	**1**	**0**	**16**
Feature of food											0 (0%)
Habit			1 (13%)	1 (50%)							2 (13%)
Index			2 (25%)								2 (13%)
Mixed food											0 (0%)
Nutrient food source				1 (50%)							1 (6%)
Other substance		1 (50%)									1 (6%)
Sugar		1 (50%)	2 (25%)		1 (100%)		2 (100%)		1 (100%)		7 (44%)
Supplement				1 (50%)							1 (6%)
Timing			1 (13%)								1 (6%)
Type of food			2 (25%)								2 (13%)
Variety of food			2 (25%)				1 (50%)				3 (19%)

The percentage is calculated based on the proportion of articles investigating each dietary variable relative to the total number of articles within the same dietary dimension (nutrient, food group, dietary pattern, and other dietary dimensions).

**Table 5.** Frequency of Adjustment Variables Utilized in the Research Articles on Breastfeeding Outcomes

Category	Variable for adjustment	Allergy	Anthropometry	Breastmilk	Development	Diet habit	Disease	Gene	Growth	Infant diet	Memory	Mental health	Metabolic	Microbiome	Micronutrient status	Neurologic	Total
Maternal demographics	Mother age	3	13	21	2		4	2	5	3	1	4	1		6	1	66
	Sociodemographic status	3	14	13	3		3	1	5	3	1	4	1		4		55
	Ethnicity		4		1		3	1	1			2			1		13
	Income		5				2		2								9
	Marital status		4				1		1		1	1					8
	Urban/rural	3		2			1		1								7
Maternal anthropometrics & body composition	Body mass index		7	19			2	2	3	2	1		1		4		41
	Gestational weight gain		5	3			2	1	4			1	1				17
	Height		3	1					2						2		8
	Pre-pregnancy BMI		1	3			1		2					1			8
	Weight		1	3									1		2		7
	Postpartum weight retention		1	1											1		3
	Body composition			1													1
Maternal health & medical factors	Parity		5	10	1		1		2		1	2			5		27
	Gestational age	3	2	4	2				1	1							13
	Mode of delivery	2	3	2			1		1	1			1				11
	Medical problems in pregnancy			1			2		1			1	1		1		7
	Maternal allergy	4															4
	Blood glucose		1						1				1				3
	Blood pressure			1													1
	Contraceptive use														1		1
Breastfeeding & lactation	Breastfeeding status		11	2			1		4	1	1		2		2		24
	Lactation stage		1	5									1				7
	Breastfeeding duration	3		1			1		1						1		7
	Breastmilk collection time			2											1		3
	Breastmilk volume			1											1		2
Infant-related factors	Infant age			8	2					2							12
	Infant sex	2		3	3				2	2							12
	Infant birth weight	2		3	1		1		3	1			1				12
	Prematurity			1													1
	Infant weight			1													1
	Birth length								1								1
	Infant allergy	1															1
Lifestyle & environmental factors	Smoking status	3	5	1	1		1	2	2	1		1	1				18
	Physical activity		8	1			1	1	2			1	1		2		17
	Alcohol use		1				1		1						1		4
	Season	2													1		3
	Sanitation & hygiene								1								1
	Sleep quality		1														1
	Lifestyle										1						1
Nutritional intake	Energy intake	2	10	10	1		2		6		1		3		2		37
	Fat intake		2	3					1								6
	Supplement use			4					1								5
	Carbohydrate intake		1	2													3
	Protein intake			2					1								3

## RESULTS

**Figure 2.**
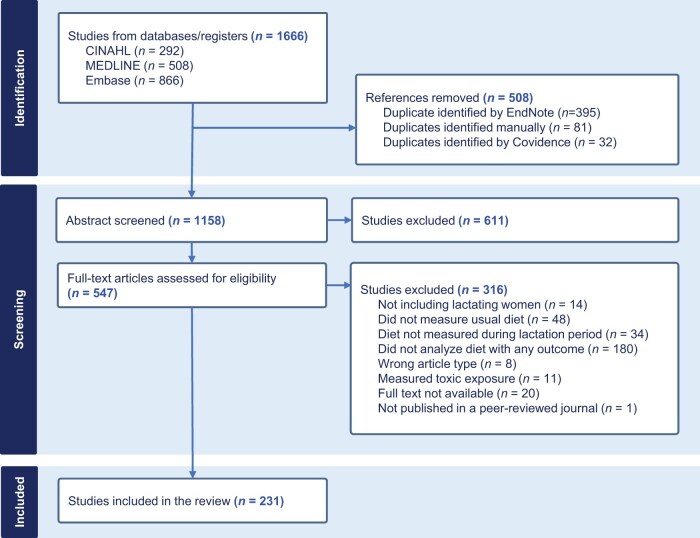
Identification, Screening, and Inclusion Criteria using PRISMA-ScR Methodology

The PRISMA-ScR (Preferred Reporting Items for Systematic Reviews and Meta-Analyses Extension for Scoping Reviews) methodology guided the scoping review process, starting with identifying 1666 relevant studies across various databases (Figure 2 and Table S6). Following thorough screening procedures, which involved removing references and identifying duplicates, 1158 abstracts were carefully examined. Subsequent to this screening phase, 611 studies were excluded based on predetermined criteria. A full-text assessment was then conducted on 547 articles to ascertain eligibility, excluding 316 studies that did not align with the study’s scope. These exclusions were based on factors such as the absence of lactating women, diet not measured during lactation, or the lack of relevant outcomes analysis. Ultimately, 231 studies were deemed suitable for inclusion in the review. Full citations of the included articles can be found in Supplementary Material S1.

### Characteristics of the Studies Included in the Scoping Review

**Figure 3.**
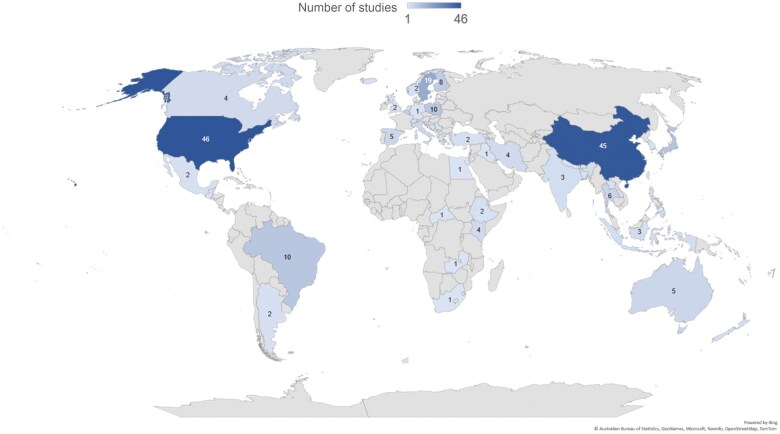
Distribution of Research on Women’s Diets and Breastfeeding Outcomes

The scoping review results highlight several key findings regarding research on lactating women’s diet and breastfeeding outcomes. First, the research has predominantly been conducted in high-income and upper-middle-income countries (HICs and UMICs), with the United States (46 studies) and China (45 studies) leading the way, followed by Sweden (19 studies), and Japan (11 studies), and then Brazil, Poland, Finland, and Thailand (these later countries had 10 studies in total; see Figure 3). Second, the geographical distribution indicates that most studies have been conducted in subtropical countries, with very few originating from African countries and none from Russia, despite its large land area and population. Notably, only a quarter of all countries in the world have participated in studies related to maternal diet during lactation and breastfeeding outcomes.

Table 1 shows the characteristics of the studies included in the scoping review. The most prevalent methods used to assess dietary intake among lactating women were Food Frequency Questionnaires (FFQs) and Food Recall (Table 1 and Supplementary Dataset).^26^ The study design predominantly consisted of cross-sectional studies (64%), followed by cohort studies (27%). Moreover, there was a noticeable scarcity of research on longer lactation durations beyond 6 months, with only 17% of studies extending for up to 1 year, 14% up to 2 years, and just 2% extending beyond 2 years of infant age. The inclusion criteria typically required participants to be breastfeeding only 1 child (26%), to have delivered a full-term infant (24%), and to be currently exclusively breastfeeding (18%). Other inclusion criteria specified being within a certain postpartum period, being able to read and speak English, having a normal body mass index (BMI), having a parity of 3 or fewer, avoiding estrogen-containing contraceptives, reporting good breast health (no cracked or bleeding nipples; no mastitis), having another child aged 2–5 years, and having had a natural or spontaneous delivery. Common exclusion criteria included having known medical problems or chronic diseases (55%) and smoking (21%). Other exclusion criteria included having particular dietary habits such as spontaneous intake restrictions, vegetarianism, avoiding dietary components due to known food allergies, using assisted reproductive technology, not being first-time mothers, providing an insufficient breast milk sample, using contraceptive medication after birth, having infants or toddlers consuming special diets or having developmental delays, and becoming pregnant again within 1 year postpartum.

### General Distribution of Diet–Outcome Analyses

Table 2 presents the number of statistical analyses conducted based on various dimensions of diet and their corresponding outcomes for both mothers and children. For dietary dimensions, the majority of analyses (53%) focused on nutrient-based dietary dimensions, while only 6% focused on dietary patterns. With respect to outcomes, analyses related to mothers represented the majority at 88%, while those related to children accounted for only 12% of the total analyses. Further analysis revealed that, among all analyses with outcomes in mothers, a significant portion (78%) pertained to breast milk composition (Table 3). Consequently, only 20% of the total analyses conducted on diet in lactating women with breastfeeding outcomes focused on outcomes specific to mothers.

### Landscape of Research in Lactating Women’s Diet in Relation to Breastfeeding Outcomes

When exploring the association between dietary variables in lactating women and breastfeeding outcomes, it became apparent that research attention has varied across different aspects. A significant discrepancy in research coverage was observed, especially concerning infant outcomes.

Dietary investigations related to maternal outcomes have predominantly revolved around nutrient intake. Researchers have focused on energy, macronutrients, fatty acids, trace minerals, and vitamin A levels. Additionally, specific food groups such as fish, vegetables, and meat, along with predefined dietary patterns, such as those associated with healthy or Mediterranean diets, have been topics of interest. Researchers have also explored other dietary variables, including the type and diversity of food consumed, to understand their impact on maternal health during lactation.

Studies on maternal outcomes have given significant attention to breast milk composition, micronutrient status, and anthropometric measurements (Table 3). Mental health– and microbiome-related outcomes have also garnered considerable interest. However, areas such as metabolic parameters, genetic influences, memory/cognitive function, and appetite regulation, have remained relatively underexplored in the current literature.

Dietary variables studied in relation to infant outcomes have encompassed maternal fatty acid intake, fruit consumption, and sugar intake. In terms of infant outcomes, primary research attention has been directed toward growth parameters and allergy development, followed by investigations into child dietary habits and overall child development (Table 4). Fewer studies have examined outcomes such as micronutrient status, genetics, and metabolic markers in children.

Overall, this analysis highlights the diverse landscape of the research concerning dietary variables in lactating women and their implications for breastfeeding outcomes. While certain areas have received significant attention, others have remained relatively underexplored, suggesting avenues for further investigation and indicating potential gaps in the current knowledge.

### Adjustment Variables in Breastfeeding Outcome Analyses

In Table 5, adjustments made during the analysis of breastfeeding outcomes are delineated, providing insight into the pivotal variables and their prevalence as covariates. Maternal age and socio-economic status (SES), such as occupation and education, emerged as the most commonly employed adjustment factors across analyses, reflecting their recognized significance in influencing breastfeeding outcomes.

Among demographic and lifestyle factors, marital status, income, physical activity, and tobacco exposure were commonly used as adjustments. However, variables such as food security, food assistance, degree of urbanicity, and alcohol exposure were less frequently considered. Including these factors in research could provide a more complete view of the influences on breastfeeding outcomes, revealing additional barriers to and facilitators of healthy outcomes, and leading to more targeted interventions. Nutritional intake variables frequently used as covariates included “background diet” factors, such as energy intake. However, background diet encompasses not only the quantity of intake but also dietary patterns, food types, and frequency of intake. Supplement use has rarely been accounted for in most outcome analyses, for mothers or children, except for breast milk composition and infant growth. This is noteworthy, given that supplementation is common during lactation, and it could introduce bias if not properly considered. Specifically, focusing on breast milk composition, the analyses frequently incorporated age, SES, parity, BMI, infant age, sex, and weight as covariates. In contrast, hormonal contraceptive use and lifestyle factors were notably absent from analyses of maternal anthropometric and metabolic outcomes.

Variables related to environmental factors (eg, sanitation, hygiene, morbidity, infection, inflammation markers) have infrequently been used in the adjustment of micronutrient status or infant growth analyses. Adjustments for inflammation are crucial when analyzing biomarkers for iron (ferritin), zinc, and vitamin A, as inflammation can alter blood levels of these nutrients.^27^ Additionally, inflammation can impede growth, as outlined in the WHO framework.^28^ Studies focusing on child outcomes continued to integrate maternal aspects, such as growth and development, and the mother’s age and SES persisted as commonly considered adjustment factors.

## DISCUSSION

This scoping review reveals a prominent gap in the exploration of diet analyses in lactating women in relation to breastfeeding outcomes, particularly child-related outcomes. Notable is the absence of research examining maternal intake of minerals and trace minerals in relation to child growth and micronutrient status, despite the understanding that certain minerals are pivotal for growth. Another example is the relationship between primary food groups, such as meat, eggs, vegetables, and fish, and their impact on both child growth and child micronutrient status. These untapped research areas present opportunities to deepen understanding of the intricate relationship between maternal diet during lactation and its ramifications for both maternal and child health outcomes, and increased knowledge of these areas is essential for formulating recommendations for this group.

### Global Distribution of Research

This scoping review also highlights a significant concentration of research into the diets of lactating women and breastfeeding outcomes in high-income countries, with minimal representation from LICs and LMICs. This imbalance limits the generalizability of existing evidence globally. This bias towards affluent nations raises concerns due to differences in food consumption, processing methods, environmental factors, and food security infrastructure between high-income and low-income settings.^29^ Studies focused on high-income countries may overlook the unique challenges faced by women in low-income contexts, such as limited access to nutritious foods and healthcare services.

Geographic location can influence the nutritional needs and status of adults, but the evidence for specific impacts on lactation performance remains limited. For example, adult populations in Asia typically have a higher percentage of body fat, which can affect metabolic health and breast milk production.^30^^,^^31^ In tropical regions with consistent sun exposure, vitamin D synthesis tends to be more robust, while temperate regions with winter seasons or colder regions may have higher caloric needs or more efficient food metabolism for energy and fat storage.^32^ Endemic diseases, such as malaria in tropical areas, can lead to chronic anemia, affecting iron levels and overall nutrition.^33^ Additionally, high-altitude regions, where lower oxygen levels stimulate increased red blood cell production, can create a higher demand for iron and other nutrients involved in hemoglobin production,^34^ potentially exacerbating anemia risks.

### Methodological Considerations

The choice of dietary assessment method depends on the study’s objectives, as discussed by Gibson.^35^ A single 24-hour recall suffices for determining average intake, whereas repeated observations on individuals or a representative subset are necessary to gauge insufficient intake prevalence. For assessing usual intake within a group, multiple 24-hour recalls, or 1-day food records are crucial, with more repetitions per individual required for statistical analysis. Within the scope of this review, in which the aim was to evaluate usual intake for correlation and regression analysis of the diet–health relationship, multiple replicates of 24-hour recalls, or 1-day food records, were essential for obtaining a detailed picture of intake. Of the 231 studies included in this review that analyzed diet outcomes, 24 studies utilized multiple replicates of 24-hour recalls, and 11 studies employed at least 1 day of food records.

The widespread use of FFQs and Food Recall methods for assessing dietary intake is notable. However, it is worth noting that in low-income countries, where context-specific FFQs may be lacking or unsuitable for the study setting, it might be more practical to increase the number of repeated 24-hour recalls per participant, instead of developing, validating, and collecting dietary data using an FFQ, as discussed by Tooze.^36^ This approach was adopted by 9 studies in LMICs and LICs in this review.^37–45^

Moreover, researchers can incorporate objective measures, such as dietary biomarkers, to validate self-reported dietary intake data. Furthermore, thorough training and standardized data collection protocols can minimize dietary assessment errors.^46^ Participants should receive clear instructions to ensure accurate reporting of portion sizes, food choices, and meal timings. Despite these efforts, it is essential to acknowledge the inherent limitations of self-reported dietary data. Factors such as social desirability bias, memory lapses, and under-reporting or over-reporting of food intake can affect data quality. Therefore, researchers should interpret the findings cautiously, considering the potential biases and limitations associated with self-reported dietary data. Sensitivity analyses and subgroup analyses can help assess the robustness of findings and identify potential sources of bias.

### Duration of Lactation

The predominance of research studies focusing on shorter lactation durations represents a notable limitation in understanding the enduring effects of maternal diet on maternal and child health outcomes. Prolonged breastfeeding has been linked to a multitude of health benefits for both mother and child. However, this review indicates that research efforts have primarily concentrated on the early postpartum period. Longitudinal studies that track maternal dietary patterns and health outcomes over extended lactation durations are indispensable for elucidating the long-term impact of maternal nutrition on maternal and child health trajectories. Such investigations can offer valuable insights into the sustained effects of maternal diet on health outcomes beyond the immediate postpartum period, thereby informing more comprehensive strategies for promoting maternal and child health throughout the lactation continuum.

### Specific Dietary Variables

The review documents several specific dietary variables, including meal frequency and timing. Three studies have explored meal frequency in a limited capacity, examining its association with maternal weight, infant weight, and breast milk vitamin E levels.^47–49^ The importance of understanding the effects of meal frequency on these outcomes is highlighted by the DGA emphasis on advancements in dietary patterns research, particularly regarding when foods are consumed throughout the day. Only 1 study has directly informed DGA recommendations regarding meal frequency during lactation and postpartum weight loss.^50^ However, further investigation into the effects of meal frequency, particularly in lactating women, is warranted, as evidenced by its relevance in other populations.^51^^,^^52^

Nutrient intake from various food sources, such as iron from animal versus plant products, has been analyzed in several studies. These analyses have examined the impact of food source on various factors, including nutrient composition in breast milk, microbiome composition, maternal risk of disease, and child allergies.^53–57^ This approach allows us to differentiate between the effect of intake of nutrients from various foods sources in which they have differing bioavailability. Additionally, the role of ultra-processed foods (UPFs) in the maternal diet and its implications for breastfeeding outcomes merit attention. Two studies analyzed UPF intake in relation to breast milk composition and weight changes.^58^^,^^59^ While not extensively studied in the context of lactation, understanding the impact of UPFs on maternal and child health outcomes could provide valuable insights into optimizing dietary recommendations for lactating women.

### Underexplored Variables

Several potential dietary variables remain underexplored in terms of their associations with maternal outcomes. These include the impact of carbohydrate derivatives on BMI, the impact of fiber on micronutrient status, and the role of minerals in cognitive/memory outcomes. In terms of food group dimension, the metabolic impacts of any foods during lactation, fruit’s influence on micronutrient status, and the effects of snack consumption on BMI and micronutrient status among lactating mothers have yet to be thoroughly investigated. The impact on metabolic markers of any dietary pattern remains to be explored.

Research aimed at understanding homeostatic processes in lactating women is particularly needed, especially regarding how higher nutrient intake affects metabolism and excretion beyond breast milk. Furthermore, the association between dietary indices and breast milk composition, as well as the effects of sugars and UPFs on metabolic markers during lactation, warrant further investigation.

Child outcomes in relation to various dietary dimensions and variables present a rich area for exploration. Within the nutrient dimension, the effects of amino acids, minerals, trace minerals, energy intake, fatty acids (such as AA and DHA), proteins, fats, carbohydrates, and vitamins in the diet of lactating mothers could be explored in relation to diverse child outcomes. These outcomes range from overall growth and metabolic markers to specific areas, such as child development and micronutrient status. Associations between foods (such as eggs, beans, nuts, fish, meat, and vegetables) and child outcomes (such as growth, micronutrient status, allergy-related outcomes, and metabolic markers) also offer avenues for investigation. Additionally, research on dietary patterns in lactating women and their impact on child outcomes is needed to inform future dietary guidelines, as emphasized by guideline committees, particularly in the United States.^20^^,^^23^^,^^24^ The effects of additional variables, such as dietary diversity, nutrient intake from various food sources, supplementation, and food variety, also need to be explored in terms of child outcomes, including allergic responses, micronutrient status, and developmental trajectories, respectively.

This review has identified several outcomes that have received relatively less exploration, including children’s metabolic markers, child developmental trajectories, and metabolic issues for mothers during lactation. Lastly, understanding the intricate dynamics of the triad encompassing the mother, breast milk, and the infant is paramount. Research elucidating nutritional epidemiology in lactation is crucial for clarifying the nutritional pathways between mother and child. Simultaneous investigations into maternal nutrient intake, maternal micronutrient status, nutrient concentrations in breast milk, infant micronutrient status, and the longitudinal growth of infants are needed in order to comprehensively delineate these pathways, and to provide a holistic understanding of the nutritional requirements of both mother and child and facilitate the development of more robust and informed nutritional recommendations.

### A Priori and a Posteriori Approaches in Dietary Analyses

Two primary methods are commonly employed to evaluate dietary intake. The first method involves assessing the diet in relation to predetermined criteria, known as “a priori” dietary patterns. These criteria may include national or global dietary recommendations. The second method, known as “a posteriori” dietary patterns or “exploratory” approaches, relies on data-driven analysis to explore any outcome or to identify clusters of dietary characteristics within a given study sample.^60^

Most dietary analyses in this review were conducted a posteriori, accounting for 85% of all analyses. There were relatively few studies that analyzed diet a priori, such as in relation to outcomes based on adherence to established standards, whether at the local or global level. Assessing the proximity of maternal nutrient intake to recommended levels (%RNI) in relation to breastfeeding outcomes is crucial for evaluating the established standards within lactating populations. Furthermore, evaluation across other dimensions of the diet, such as food group intake based on local or global guidelines, is essential. A previous review on adherence to dietary guidelines for pregnant and lactating women noted a dearth of studies analyzing adherence to dietary guidelines during lactation, despite the existence of studies focusing on pregnancy.^61^ However, 14 studies in this scoping review measured dietary diversity as absolute number of different food groups consumed, using the indicator set in Minimum Dietary Diversity for Women (MDD-W), or, in earlier studies, the previous indicator, the Women’s Dietary Diversity Score (WDDS).^39^^,^^40^^,^^44^^,^^47^^,^^62–72^ These indicators serve as global dietary recommendations for women of reproductive age endorsed by the FAO and FHI360 and have demonstrated efficacy in measuring micronutrient adequacy.^73^ Among these studies, MDD-W was analyzed in relation to various outcomes, including maternal mid-upper arm circumference (MUAC), mental health (anxiety and depression), maternal micronutrient status (vitamin A, folate, vitamin B12, and anemia), breast milk composition, child growth, and child dietary habits.^39^^,^^40^^,^^68^^,^^72^ None of the studies analyzing outcomes related to dietary quality in lactating women used other indices such as (1) the Global Dietary Recommendations (GDR) score, which reflects WHO recommendations for healthy diets, (2) NOVA, which serves as a proxy for the dietary share of UPFs, or (3) the Global Diet Quality Score (GDQS), which measures the consumption of food groups that contribute to nutrient adequacy and reduce the risk of noncommunicable diseases (NCDs).

### Strengths and Limitations

The strengths of this scoping review lie in its comprehensive search strategy, which captures a broad range of articles without restricting outcomes. This approach has enabled an extensive overview of the research in this field and indicated numerous variables that have not been adequately studied. However, several limitations should be noted. First, the exclusion of articles that did not conduct dietary assessments may have resulted in the omission of relevant clinical trials, particularly those involving nutritional interventions. This exclusion, designed to enable the compiling of dietary variables from multiple assessments, may have overlooked studies focusing solely on intervention levels without capturing information on usual dietary intake. Second, the review’s inclusion criteria specifying articles in English may have introduced language bias and excluded relevant studies published in other languages. Finally, due to the extensive processing time, the review may have overlooked publications from 2023 onward, as the latest articles included were from the end of 2022. These limitations should be considered when interpreting the findings of this scoping review.

## CONCLUSION AND RECOMMENDATION

In summary, this review identified significant gaps in the current research on dietary factors for lactating women and maternal and child breastfeeding outcomes, especially in low-income settings. Methodologically, more a priori analyses and longitudinal studies are needed in order to understand the long-term effects of maternal diet on both maternal and child health. Additionally, when analyzing diet–health relationships, improving dietary assessment methods to accurately capture usual intake and minimize self-reporting bias is crucial. Under-researched dietary and outcome variables present opportunities for further research that will enhance our understanding of the links between maternal diet and breastfeeding outcomes, and thus support development of the dietary guidelines.

## Supplementary Material

nuae228_Supplementary_Data

## Data Availability

The data^26^ underlying this article are available in Figshare at 10.6084/m9.figshare.25964137.
