# Supplementary material for: A Scoping Review of Methodologies Exploring Diet and Health Outcomes in Lactating Women: What Has Been Done and Where to Next?
Source: Nutr Rev. 2025 Feb 17;83(9):1808–25. doi: 10.1093/nutrit/nuae228 (PMC12343025; doi:10.1093/nutrit/nuae228)
Supplement: nuae228_Supplementary_Data [file nuae228_supplementary_data.docx]

Supplementary Material

**Exploring Diet and Health Outcomes in Lactating Women: What Has Been Done and Where to Next? A Scoping Review**

## Supplementary Table 1. Nutrient-based variables

| **Nutrient** | **Variables (unit)** |
| --- | --- |
| Energy | energy (kcal/d), (kcal/kg body weight) |
| Macronutrient  (Energy-yielding nutrient) | carbohydrate, protein, fat (g/d), (%energy)  categorisation: above/below range, adequate |
| Carbohydrate derivatives | fructose, disaccharides, lactose (g/d) |
| Amino acid | amino acid (%/d)  lysine, isoleucine, threonine, valine, serine, methionine, cysteine, phenylalanine, and tyrosine (mg/g protein)  carnitine (mmol/d) |
| Fatty acid | SAFA/SFA, MUFA, PUFA, trans-FA (g/d), (%fat), (%energy)  MUFA/PUFA ratio, MUFA/SAFA ratio, PUFA/SAFA ratio  total fatty acid, essential fatty acid (EFA), linoleic acid (LA) or omega-6, alpha linolenic acid (ALA) or omega-3, docosahexaenoic acid (DHA), docosapentaenoic acid (DPA), eicosapentaenoic acid (EPA), cholesterol, stearic, pentadecanoic (g/d), (mg/d), (%/d)  omega6/omega3 ratio |
| Fibre | Fibre (g/d), (g/1000 kcal)  soluble, insoluble (g/d) |
| Vitamin A | vitamin A (mcg/d), (RE mcg/d), (RAE mcg/d)  retinol, beta-carotene, beta-cryptoxanthin, carotenoid (mg/d)  lutein, lycopene (mg/d), (times/d) |
| Vitamin B | thiamine, riboflavin, niacin, pantothenic acid, vitamin B6, folate, vitamin B12, choline (mg/d), (mcg/d) |
| Vitamin C | Vitamin C (mg/d), (high/medium/low) |
| Vitamin D | Vitamin D (mcg/d), (DFR mg/d), (poor/fair)  Vitamin D2, D3 (IU/d) |
| Vitamin E | Vitamin E (mg/d), tocopherol (mcg/d), vitamin E/PUFA ratio |
| Mineral | Calcium, iron, magnesium, phosphorus (mg/d)  Iron (high/medium/low), magnesium density (mg/1000kcal) |
| Trace mineral | Zinc, manganese, selenium, chromium, molybdenum, iodine, fluoride, cooper (mg/d), (mcg/d) |
| Electrolyte | sodium, potassium (mg/d), sodium chloride (g/d) |
| Polyphenol | Total polyphenol, anthocyanin, epicatechin, phenolic acid, flavanone, flavonoid, flavanol, flavan-3-ol, isoflavone (mg/d) |
| Other | Alkaloid: caffeine, theobromine, theophylline (mg/d)  phenolic compound: tyrosyl (mg/d)  phytonutrient: lignan (mg/d)  antinutrient: phytate (mg/d) |

## Supplementary Table 2. Food-based variables

| **Food** | **Variables (unit)** |
| --- | --- |
| bean and nut | beans (g/d), fresh soybean, legumes, nuts, soy products (serving/d) |
| dairy | cheese (g/d), cow milk (ml/d, ml/w, serving/d), dairy, dairy-based dessert, milk (any) |
| drink | coffee (serving/m, serving/w), tea (serving/d) |
| egg | egg, any (g/d, oz/d, pcs/d, pcs/w, serving/d, serving/w)  egg, whole (serving/w), salted egg (serving/d) |
| fish | fish, any (g/d, serving/d, serving/w), fish, fatty (serving/w, g/d)  fish, fresh (serving/w) fish, canned (serving/w)  fish, saltwater (g/d), fish, freshwater (g/d)  fish, grilled (g/1000 kcal), fish, lean (g/d)  fish, mandarin (g/d), fish, raw, boiled, deep fried (g/1000 kcal)  fish, salmon (g/d), fish and shrimp (g/d, score), salted fish (serving/d) |
| fruit | fruit, any (%, cup/d, g/d, score), fruit, whole (serving/d, score)  fruit juice (serving/d, serving/w)  fruit and berry (g/d, serving/d), apple (serving/w)  banana flower, basil, gourd (serving/w), probiotic (score, serving/d, serving/m) |
| grain | grain, any (g/d, serving/d, yes/no)  grain, whole (oz/d, serving/d) and grain, non-whole grain (serving/d)  grain, total (serving/d)  rice (serving/d), bread (g/d), cereal (g/d, serving/d, serving/w), doughnut (serving/w) |
| herb | hot chilli sauce (serving/d), condiment and seasoning (serving/d),  ginger vinegar (yes/no) |
| meat | meat, any (g/d, serving/d, serving/w, serving/m)  game meat and processed meat (g/d), smoked meat (serving/d)  beef (g/d), chicken/poultry (g/d)  meat and eggs (serving/d), meat or poultry (g/d) |
| mixed food | dairy, egg, fish, shrimp (g/d), milk and meat (yes/no), red meat and vegetable (g/d), seafood and plant protein intake (score), dairy and pizza (serving/d) |
| fat and oil | fats and oils, any (serving/d), linseed and coconut oil (g/d)  butter (g/d), margarine (g/d), margarine and oils (g/d) |
| snack | bakery, confectionery, snack (serving/d), candy (pcs/d) |
| specific food | pickles food (serving/d), Chinese cabbage (serving/w), croaker (g/d)  insect and small rodent (serving/d)  natto: traditional Japanese food (mg/d), enset: traditional Ethiopian food (serving/d) |
| vegetable | vegetable (g/d, serving/d)  *no specific vegetable is being explored* |

## Supplementary Table 3. Dietary patterns

| **Dietary Pattern** | **Variables (unit)** |
| --- | --- |
| dominant food – a pattern that is defined by the dominant food group consumed | cereal and sweet, fruit and vegetable, plant food, soup, vegetable, and fruit, starch and vegetable protein, high-energy snack and processed food, marine and flour  Chinese confinement, Indian confinement, traditional northern |
| dominant nutrient - pattern that is defined by the dominant nutrient intake | high fat (%), low fat (%), high protein (%), low protein (%)  fatty acid with fin, fatty acid with leave, fatty acid with leg  high SAFA, low vitamin C |
| pre-defined pattern | healthy (tertile), unhealthy (tertile), Mediterranean, my pyramid |

## Supplementary Table 4. Other dietary dimensions

| **Other** | **Variables (unit)** |
| --- | --- |
| feature of food | glycemic index, glycemic load (/1000 kcal)  acid load (x10 mEq/d), nutrient index (%) |
| habit | amount of food (less/same/more compared to other period),  diet avoidance, eating out, dietary restriction (yes/no)  meal size (kcal/meal) |
| index | Alternate Mediterranean Diet index (aMED) (high/low score, score)  Dietary Inflammatory Index (DII) (score), Puerperium Energy-adjusted DII (P-EDII) (score, quantiles)  Healthy Eating Index (HEI), HEI-2005, HEI-2015, AHEI-2010 (score), HFP (score)  Low carb diet – (LCD), plant LCD, animal LCD (score)  Plant-based Diet Index (PDI) (score), healthful PDI (hPDI), Unhealthful PDI (uPDI) (score)  total diet quality (score) |
| nutrient food source | DHA from fish, freshwater (g/d), EPA from fish, freshwater (g/d)  protein from animal (g/d), vitamin A from animal, vitamin A from plant (mcg/d)  vitamin C from food, vitamin C from supplements (mg/d)  vitamin D from food, vitamin D from supplements (mcg/d)  iodine from food, iodine from supplements (mg/d)  epicatechin from cocoa (mg/d)  nitrite from soybean product, nitrite from other foods (serving/d)  nitrosamines from soybean product, nitrosamines from other food (serving/d)  fibre from grain (g/d) |
| other substance | alcohol (%energy, oz/d) |
| sugar | sugar, added sugar (g/d, tsp/d, g/1000kcal, %energy, serving/d)  SSB (g/d, oz/d, serving/d, serving/m, %energy)  discretionary energy (g/d), empty calories (score)  sweets (%), syrup and sweet sauce (serving/d) |
| supplement | Supplement, any (yes/no)  cod liver oil (yes/no), fish oil (yes/no)  vitamin D, folic acid (mcg), iron (g/d), selenium (yes/no), DHA (times/w) |
| meal occasion | breakfast (times/w), meal frequency (times/d, <3,3-4,>4/d) |
| type of food | animal-source food (ASF) (g/d, score, item/d, %RNI)  protein food (type, serving/w), fried food (serving/w),  hypaphorine-containing foods: lentils, chickpeas, peanuts (g/d)  probiotic, boiled root crops (times/w), probiotic, corn chips (times/w)  galactagogues (kind, frequency, and amount)  table salt, added to meal (yes/no), UPF (%, %energy z-score)  water (ml/d, adequate/low), drinks (times/d) |
| variety of food | Dietary diversity score (DDS), Women DDS (WDDS), Minimum Dietary Diversity for Women (MDDW) (score)  dietary diversity (<4, 4-5, >5/d), number of different food (/3d)  number of food group, food group (>5 yes/no)  food group from the food pyramid (times/d) |

## Supplementary Table 5. Outcome Variables

| **Mother’s outcomes** |
| --- |
| **breastmilk** |
| breastmilk: AA, AA/DHA ratio, AA/EPA ratio, adrenic acid, ALA, alpha-linolenic acid, alpha-tocopherol, amino acid (total), anti-lactoglobulin, anti-ovalbumin, ARA, ARA:(EPA+DHA), beta carotene, beta cryptoxanthin, betaine, C16:00, C18:0, C18:2, C18:2n-6, C18:3n-3, caffeic acid, caffeine, calcium, carbohydrate, carnitine, carotenoid, chlorogenic acid, choline, copper, daidzein (DAI), DHA, DHA proportion, dihomo linolenic acid, docosate-traenoic acid, DPA, DPPH, dry matter, elaidic acid, energy, EPA, EPA+DHA, epicatechin, epicatechin metabolite, epidermal growth factor (EGF), estradiol, estriol, fat, fatty acid, ferulic acid, folate, gallic acid, gamma-tocopherol, glutamic acid, glycerophosphocholine, HMO, hypaphorine, iodine, iron, isoleusin, kaempferol, LA, LA/DHA ratio, lactoferrin, lactoglobulin, lactose, lactose+protein, lauric acid, leucine, linolenic acid, lipid, lutein, lutein+zeaxantin, lycopene, lysine, magnesium, methyxanthine, micronutrient, milk production, MUFA, MUFA/SAFA ratio, naringenin, natrium, niacin, nitrogen, omega3 FA, omega6 FA, omega6/omega 3 FA ratio, other FA, ovalbumin, palmitic acid, pantothenic acid, paraxanthine, pH, phenol, phenylalanine, phosphatidylcholine, phosphocholine, phosphorus, polyphenol, potassium, progesterone, protein, PUFA, PUFA/SAFA ratio, quercetin, retinol, riboflavin, rumenic acid, SAFA, selenium, sialic acid, sugar, theobromine, theophylline, thiamine, thiol, tocopherol, total protein, trace element, trans FA, trans fatty acid <2, trans fatty acid >4, trans fatty acid 2-4, transforming growth factor (TGF)-alpha, true protein, vaccenic acid, valine, vitamin A, vitamin A foremilk, vitamin A hindmilk, vitamin A mixed, vitamin B6, vitamin B12, vitamin C, vitamin D, vitamin E, volume, zeaxanthin, zinc  breastmilk volume  colostrum: AA, ALA, beta carotene, carotenoid, EPA, iodine colostrum, LA, MUFA, omega3 FA, omega6 FA, PUFA, retinol, SAFA |
| **anthropometry** |
| anthropometry: abdominal circumference, BMI, BMIZ, height, low MUAC, undernutrition, waist circumference, weight, body composition, body fat, fat mass, muscle mass  weight change: continuous PPWR, gestational weight gain retained, postpartum weight change, postpartum weight loss, postpartum weight retention, substantial PPWR (>=4.55 kg), weight change, weight loss, weight retention |
| **Appetite** |
| behaviour: pre-meal hunger, after-meal hunger |
| **Disease** |
| inflammation: CRP, IL-6  mother disease: puerperium health problems: risk of nasopharyngeal carcinoma (NPC)  symptom: hypohydration |
| **Gene** 🡪 statistical analysis |
| gene: DNA damage, leucocyte telomere length (LTL) |
| **memory** |
| learning, lexical-semantic memory, long-term memory, short-term memory, working memory |
| **mental health** |
| anxiety state, depression state, Edinburgh Postnatal Depression Scale (EPDS), Parenting Stress Inventory (PSI), Postpartum depression (PPD), sleep duration, State-Trait Anxiety Inventory (STAI) |
| **metabolic** |
| fasting glucose, HDL, LDL, leptin, OGTT, risk of IFG/IGT, total cholesterol, triglyceride |
| **microbiome** |
| Staphylococci, Acinetobacter, Bifidobacteria, bray-curtis, chao1, Corynebacterium, Firmicutes, Gemella, Lactobacillus, Rothia, Shannon, Staphylococcus, Streptococcus, Veilonella, Total plate count (TPC) |
| **micronutrient status** |
| blood: anemia, calcium turnover, DHA phospholipid, EPA, EPA+DPA phospholipid, folate, folate deficiency, hb and body iron, linolenic acid, magnesium, MMA, omega6, omega6/omega 3 FA ratio, retinol, selenium, total body vitamin A stores, vitamin B12, vitamin D, zinc, zinc fractional absorption, zinc homeostasis, zinc serum  eye: macular pigment optical density (MPOD)  Hair: zinc  imaging: bone calcium, bone density, bone mass, bone mineral density, change in lean soft tissue, cortical thickness, osteo sono-assessment index (OSI), trabecular thickness  RBC: DHA erythrocyte, folate in RBC, omega6 erythrocyte  urine: 24h selenium, fluoride, free carnitine, iodine, iodine 24h, iodine spot, osmolality, selenium, selenium:creatinine ratio, total carnitine |
| **Infant’s outcomes** |
| **allergy** |
| asthma, atopic eczema, atopic sensitisation, cow milk allergy (CMA), food allergy, IgA to ovalbumin, IgE to egg white or cow milk, IgE to food, IgG to ovalbumin, risk of asthma |
| **development** |
| cognitive, Griffith score, hand-eye coordination, hearing and speech, intelligence, locomotor scale, performance, social/personal, vocabulary and visual motor |
| **Disease** |
| child disease: diabetes type 1, preclinical type 1 diabetes  symptom: diarrhea, gastrointestinal, reflux, vomiting |
| **Gene** |
| gene-specific methylation |
| **Growth** |
| anthropometry: BMI for age, HAZ, LAZ, macrocephaly, overweight, skinfold thickness, stunted, underweight, waist circumference, wasted, WAZ, weight, weight-for-length trajectory from birth to 1 year, WLZ  body composition: body fat, fat-free mass, fat mass |
| **infant diet** |
| added sugar intake, balanced weaning diet, breastfed duration, ever breastfed, formula, fruit intake, high-energy snack and processed food, salty snack intake, sugar-sweetened beverages, sweet intake, vegetable, vegetables, whole grains, lean protein foods intake |
| **metabolic** |
| leptin, split proinsulin |
| **microbiome** |
| 140 gene cluster in gut microbiome, 150 gene cluster in gut microbiome, 28 gene cluster in gut microbiome, 51 gene cluster in gut microbiome, Klebsiella michiganensis, Lactobacillus paracasei, microbiome composition, Veillonella parvula |
| **micronutrient status** |
| blood: fatty acid |
| **neurologic** |
| imaging: regional brain volume, voxel-wise tissue densities |

## Supplementary Table 6. The Preferred Reporting Items for Systematic reviews and Meta-Analyses extension for Scoping Reviews (PRISMA-ScR) Checklist

| **SECTION** | **ITEM** | **PRISMA-ScR CHECKLIST ITEM** | **REPORTED ON PAGE #** |
| --- | --- | --- | --- |
| **TITLE** | | | |
| Title | 1 | Identify the report as a scoping review. | 1 |
| **ABSTRACT** | | | |
| Structured summary | 2 | Provide a structured summary that includes (as applicable): background, objectives, eligibility criteria, sources of evidence, charting methods, results, and conclusions that relate to the review questions and objectives. | 3-4 |
| **INTRODUCTION** | | | |
| Rationale | 3 | Describe the rationale for the review in the context of what is already known. Explain why the review questions/objectives lend themselves to a scoping review approach. | 4-6 |
| Objectives | 4 | Provide an explicit statement of the questions and objectives being addressed with reference to their key elements (e.g., population or participants, concepts, and context) or other relevant key elements used to conceptualize the review questions and/or objectives. | 5-6 |
| **METHODS** | | | |
| Protocol and registration | 5 | Indicate whether a review protocol exists; state if and where it can be accessed (e.g., a Web address); and if available, provide registration information, including the registration number. | NA |
| Eligibility criteria | 6 | Specify characteristics of the sources of evidence used as eligibility criteria (e.g., years considered, language, and publication status), and provide a rationale. | 9 |
| Information sources* | 7 | Describe all information sources in the search (e.g., databases with dates of coverage and contact with authors to identify additional sources), as well as the date the most recent search was executed. | 8 |
| Search | 8 | Present the full electronic search strategy for at least 1 database, including any limits used, such that it could be repeated. | 8-9 |
| Selection of sources of evidence† | 9 | State the process for selecting sources of evidence (i.e., screening and eligibility) included in the scoping review. | 9 |
| Data charting process‡ | 10 | Describe the methods of charting data from the included sources of evidence (e.g., calibrated forms or forms that have been tested by the team before their use, and whether data charting was done independently or in duplicate) and any processes for obtaining and confirming data from investigators. | 10 |
| Data items | 11 | List and define all variables for which data were sought and any assumptions and simplifications made. | 10 |
| Critical appraisal of individual sources of evidence§ | 12 | If done, provide a rationale for conducting a critical appraisal of included sources of evidence; describe the methods used and how this information was used in any data synthesis (if appropriate). | NA |
| Synthesis of results | 13 | Describe the methods of handling and summarizing the data that were charted. | 10-11 |
| **RESULTS** | | | |
| Selection of sources of evidence | 14 | Give numbers of sources of evidence screened, assessed for eligibility, and included in the review, with reasons for exclusions at each stage, ideally using a flow diagram. | Figure 2 |
| Characteristics of sources of evidence | 15 | For each source of evidence, present characteristics for which data were charted and provide the citations. | Table 1 |
| Critical appraisal within sources of evidence | 16 | If done, present data on critical appraisal of included sources of evidence (see item 12). | NA |
| Results of individual sources of evidence | 17 | For each included source of evidence, present the relevant data that were charted that relate to the review questions and objectives. | Supplementary Dataset |
| Synthesis of results | 18 | Summarize and/or present the charting results as they relate to the review questions and objectives. | 12-16, Table 2-5 |
| **DISCUSSION** | | | |
| Summary of evidence | 19 | Summarize the main results (including an overview of concepts, themes, and types of evidence available), link to the review questions and objectives, and consider the relevance to key groups. | 17-24 |
| Limitations | 20 | Discuss the limitations of the scoping review process. | 24-25 |
| Conclusions | 21 | Provide a general interpretation of the results with respect to the review questions and objectives, as well as potential implications and/or next steps. | 25 |
| **FUNDING** | | | |
| Funding | 22 | Describe sources of funding for the included sources of evidence, as well as sources of funding for the scoping review. Describe the role of the funders of the scoping review. | 26 |

## Supplementary Material 1. Included Articles

(1-231)

1. Aaltonen J, Ojala T, Laitinen K, Poussa T, Ozanne S, Isolauri E. Impact of maternal diet during pregnancy and breastfeeding on infant metabolic programming: A prospective randomized controlled study. Eur J Clin Nutr. 2011;65(1):10-9.

2. Ai Z, Jian Z, Wei W, Peiyu W, Yumei Z, Zhao A, et al. Postpartum anemia is a neglected public health issue in China: a cross-sectional study. Asia Pac J Clin Nutr. 2019;28(4):793-9.

3. Al-Hassan A, Vyas R, Zhang Y, Sisitsky M, Gagoski B, Litt JS, et al. Assessment of maternal macular pigment optical density (MPOD) as a potential marker for dietary carotenoid intake during lactation in humans. Nutrients. 2022;14(1) (no pagination).

4. Alderete TL, Wild LE, Mierau SM, Bailey MJ, Patterson WB, Berger PK, et al. Added sugar and sugar-sweetened beverages are associated with increased postpartum weight gain and soluble fiber intake is associated with postpartum weight loss in Hispanic women from Southern California. Am J Clin Nutr. 2020;112(3):519-26.

5. Amir LH, Donath SM. Maternal diet and breastfeeding: a case for rethinking physiological explanations for breastfeeding determinants. Early Human Development. 2012;88(7):467-71.

6. Amorim NCM, Silva AGCLD, Reboucas AS, Bezerra DS, Lima MSR, Medeiros JFP, et al. Dietary share of ultra-processed foods and its association with vitamin E biomarkers in Brazilian lactating women. Br J Nutr. 2022;127(8):1224-31.

7. Antonakou A, Chiou A, Andrikopoulos N, Bakoula C, Matalas A-L. Breast milk tocopherol content during the first six months in exclusively breastfeeding Greek women. Eur J Nutr. 2011;50(3):195-202.

8. Antonakou A, Skenderi K, Chiou A, Anastasiou C, Bakoula C, Matalas A-L. Breast milk fat concentration and fatty acid pattern during the first six months in exclusively breastfeeding Greek women. Eur J Nutr. 2013;52(3):963-73.

9. Aubuchon-Endsley NL, Kennedy TS, Gilchrist M, Thomas DG, Grant S. Relationships among Socioeconomic Status, Dietary Intake, and Stress in Breastfeeding Women. Journal of the Academy of Nutrition & Dietetics. 2015;115(6):939-46.e1.

10. Aumeistere L, Belusko A, Ciprovica I, Zavadska D. Trans Fatty Acids in Human Milk in Latvia: Association with Dietary Habits during the Lactation Period. Nutrients. 2021;13(9) (no pagination).

11. Aumeistere L, Ciprovica I, Zavadska D, Andersons J, Volkovs V, Celmalniece K. Impact of Maternal Diet on Human Milk Composition Among Lactating Women in Latvia. Medicina. 2019;55(5).

12. Aumeistere L, Ciprovica I, Zavadska D, Bavrins K, Borisova A. Zinc content in breast milk and its association with maternal diet. Nutrients. 2018;10(10) (no pagination).

13. Awoke MA, Wycherley TP, Earnest A, Skouteris H, Moran LJ. The Profiling of Diet and Physical Activity in Reproductive Age Women and Their Association with Body Mass Index. Nutrients. 2022;14(13) (no pagination).

14. Barennes H, Simmala C, Odermatt P, Thaybouavone T, Vallee J, Martinez-Ussel B, et al. Postpartum traditions and nutrition practices among urban Lao women and their infants in Vientiane, Lao PDR. Eur J Clin Nutr. 2009;63(3):323-31.

15. Batalha MA, Ferreira ALL, Freitas-Costa NC, Figueiredo ACC, Carrilho TRB, Shahab-Ferdows S, et al. Factors associated with longitudinal changes in B-vitamin and choline concentrations of human milk. Am J Clin Nutr. 2021;114(4):1560-73.

16. Bayaga CLT, Tanguilig KMN, Aba RPM, Pico MB, Gabriel AA. Culturable micro-organisms in human milk were found to be associated with maternal weight, diet and age during early lactation. J Appl Microbiol. 2021;131(2):925-37.

17. Belfort MB, Rifas-Shiman SL, Kleinman KP, Guthrie LB, Bellinger DC, Taveras EM, et al. Infant feeding and childhood cognition at ages 3 and 7 years: Effects of breastfeeding duration and exclusivity. JAMA Pediatrics. 2013;167(9):836-44.

18. Berg MJ, Van Dyke DC, Chenard C, Niebyl JR, Hirankarn S, Bendich A, et al. Folate, zinc, and vitamin B-12 intake during pregnancy and postpartum. Journal of the American Dietetic Association. 2001;101(2):242-5.

19. Berger PK, Plows JF, Jones RB, Alderete TL, Rios C, Pickering TA, et al. Associations of maternal fructose and sugar-sweetened beverage and juice intake during lactation with infant neurodevelopmental outcomes at 24 months. Am J Clin Nutr. 2020;112(6):1516-22.

20. Bertz F, Winkvist A, Brekke HK. Sustainable weight loss among overweight and obese lactating women is achieved with an energy-reduced diet in line with dietary recommendations: results from the LEVA randomized controlled trial. Journal of the Academy of Nutrition and Dietetics. 2015;115(1):78-86.

21. Blanchard J, Weber CW, Shearer LE. Methylxanthine levels in breast milk of lactating women of different ethnic and socioeconomic classes. Biopharm Drug Dispos. 1992;13(3):187-96.

22. Boghossian NS, Yeung EH, Lipsky LM, Poon AK, Albert PS. Dietary patterns in association with postpartum weight retention. Am J Clin Nutr. 2013;97(6):1338-45.

23. Bosha T, Lambert C, Riedel S, Melesse A, Biesalski HK. Dietary diversity and anthropometric status of mother-child pairs from enset (False banana) staple areas: A panel evidence from southern Ethiopia. Int J Environ Res Public Health. 2019;16(12) (no pagination).

24. Botros RM, Sabry IM, Abdelbaky RS, Eid YM, Nasr MS, Hendawy LM. Vitamin D deficiency among healthy Egyptian females. Endocrinol. 2015;62(7):314-21.

25. Bottin JH, Eussen SRBM, Igbinijesu AJ, Mank M, Koyembi J-CJ, Nyasenu YT, et al. Food Insecurity and Maternal Diet Influence Human Milk Composition between the Infant's Birth and 6 Months after Birth in Central-Africa. Nutrients. 2022;14(19):4015.

26. Bravi F, Di Maso M, Eussen S, Agostoni C, Salvatori G, Profeti C, et al. Dietary Patterns of Breastfeeding Mothers and Human Milk Composition: Data from the Italian MEDIDIET Study. Nutrients. 2021;13(5):19.

27. Brembeck P, Winkvist A, Bååth M, Bärebring L, Augustin H. Determinants of changes in vitamin D status postpartum in Swedish women. Br J Nutr. 2016;115(3):422-30.

28. Brembeck P, Winkvist A, Ohlsson C, Lorentzon M, Augustin H. Determinants of microstructural, dimensional and bone mineral changes postpartum in Swedish women. Br J Nutr. 2016;116(10):1736-44.

29. Buntuchai G, Pavadhgul P, Kittipichai W, Satheannoppakao W. Traditional Galactagogue Foods and Their Connection to Human Milk Volume in Thai Breastfeeding Mothers. Journal of Human Lactation. 2017;33(3):552-9.

30. Butte NF, Garza C, Stuff JE, Smith EO, Nichols BL. Effect of maternal diet and body composition on lactational performance. Am J Clin Nutr. 1984;39(2):296-306.

31. Butts CA, Hedderley DI, Herath TD, Paturi G, Glyn-Jones S, Wiens F, et al. Human milk composition and dietary intakes of breastfeeding women of different ethnicity from the manawatu-wanganui region of New Zealand. Nutrients. 2018;10(9) (no pagination).

32. Bzikowska-Jura A, Czerwonogrodzka-Senczyna A, Jasinska-Melon E, Mojska H, Oledzka G, Wesolowska A, et al. The Concentration of Omega-3 Fatty Acids in Human Milk Is Related to Their Habitual but Not Current Intake. Nutrients. 2019;11(7):12.

33. Bzikowska-Jura A, Czerwonogrodzka-Senczyna A, Oledzka G, Szostak-Wegierek D, Weker H, Wesolowska A. Maternal nutrition and body composition during breastfeeding: Association with human milk composition. Nutrients. 2018;10(10) (no pagination).

34. Bzikowska-Jura A, Sobieraj P, Michalska-Kacymirow M, Wesołowska A. Investigation of Iron and Zinc Concentrations in Human Milk in Correlation to Maternal Factors: An Observational Pilot Study in Poland. Nutrients. 2021;13(2):303-.

35. Bzikowska-Jura A, Sobieraj P, Szostak-Wegierek D, Wesolowska A. Impact of infant and maternal factors on energy and macronutrient composition of human milk. Nutrients. 2020;12(9):1-14.

36. Cahill JM, Freeland-Graves JH, Shah BS, Lu H, Pepper MR. Determinants of weight loss after an intervention in low-income women in early postpartum. Journal of the American College of Nutrition. 2012;31(2):133-43.

37. Cai X, Duan Y, Li Y, Wang J, Mao Y, Yang Z, et al. Lactoferrin level in breast milk: a study of 248 samples from eight regions in China. Food & Function. 2018;9(8):4216-22.

38. Carbone P, Sobreviela M, Jimenez D, Martinez C, Pocovi M. Hair zinc and dietary zinc intake during pregnancy and puerperium. European Journal of Obstetrics Gynecology and Reproductive Biology. 1992;47(2):103-8.

39. Castiblanco-Rubio GA, Munoz-Rocha TV, Tellez-Rojo MM, Ettinger AS, Mercado-Garcia A, Peterson KE, et al. Dietary Influences on Urinary Fluoride over the Course of Pregnancy and at One-Year Postpartum. Biological Trace Element Research. 2022;200(4):1568-79.

40. Castro I, Arroyo R, Aparicio M, Martinez MA, Rovira J, Ares S, et al. Dietary habits and relationship with the presence of main and trace elements, bisphenol a, tetrabromobisphenol a, and the lipid, microbiological and immunological profiles of breast milk. Nutrients. 2021;13(12) (no pagination).

41. Cena H, Castellazzi AM, Pietri A, Roggi C, Turconi G. Lutein concentration in human milk during early lactation and its relationship with dietary lutein intake. Public Health Nutr. 2009;12(10):1878-84.

42. Chan SM, Nelson EA, Leung SS, Li CY. Postnatal iron status of Hong Kong Chinese women in a longitudinal study of maternal nutrition. Eur J Clin Nutr. 2001;55(7):538-46.

43. Cummings JR, Lipsky LM, Schwedhelm C, Liu A, Nansel TR. Associations of ultra-processed food intake with maternal weight change and cardiometabolic health and infant growth. Int J Behav Nutr Phys Act. 2022;19(1) (no pagination).

44. Cunha LR, Costa TH, Caldas ED. Mercury concentration in breast milk and infant exposure assessment during the first 90 days of lactation in a midwestern region of Brazil. Biological Trace Element Research. 2013;151(1):30-7.

45. da Silva A, de Sousa Reboucas A, Mendonca BMA, Silva D, Dimenstein R, Ribeiro K. Relationship between the dietary intake, serum, and breast milk concentrations of vitamin A and vitamin E in a cohort of women over the course of lactation. Matern Child Nutr. 2019;15(3):e12772.

46. Dai X, Yin H, Zhang J, Tian F, Cai X, Mao Y, et al. Carotenoid Profile in Maternal/Cord Plasma and Changes in Breast Milk along Lactation and Its Association with Dietary Intake: A Longitudinal Study in a Coastal City in Southern China. Nutrients. 2022;14(9) (no pagination).

47. Daneshzad E, Moradi M, Maracy MR, Brett NR, Bellissimo N, Azadbakht L. The association of maternal plant-based diets and the growth of breastfed infants. Health Promotion Perspectives. 2020;10(2):152-61.

48. Daniels L, Gibson RS, Diana A, Haszard JJ, Rahmannia S, Luftimas DE, et al. Micronutrient intakes of lactating mothers and their association with breast milk concentrations and micronutrient adequacy of exclusively breastfed Indonesian infants. Am J Clin Nutr. 2019;110(2):391-400.

49. Davis JN, Shearrer GE, Tao W, Hurston SR, Gunderson EP. Dietary variables associated with substantial postpartum weight retention at 1-year among women with GDM pregnancy. BMC Obesity. 2017;4(1) (no pagination).

50. De Carvalho Souza MDD, Mary Ribeiro M, Bueno Ferreira L, Silva Do Carmo A, Dos Santos LC. Weight Reduction and Changes in Body Circumferences in Lactating Women as a Function of Differences in Dietary Macronutrient Content. Breastfeeding Medicine. 2022;17(6):511-8.

51. de Castro MB, Kac G, de Leon AP, Sichieri R. High-protein diet promotes a moderate postpartum weight loss in a prospective cohort of Brazilian women. Nutrition. 2009;25(11-12):1120-8.

52. de Castro MB, Sichieri R, Barbosa Brito Fdos S, Nascimento S, Kac G. Mixed dietary pattern is associated with a slower decline of body weight change during postpartum in a cohort of Brazilian women. Nutricion Hospitalaria. 2014;29(3):519-25.

53. Demmelmair H, Kuhn A, Dokoupil K, Hegele V, Sauerwald T, Koletzko B. Human lactation: oxidation and maternal transfer of dietary (13)C-labelled alpha-linolenic acid into human milk. Isotopes in environmental and health studies. 2016;52(3):270-80.

54. Deng L, Zou Q, Liu B, Ye W, Zhuo C, Chen L, et al. Fatty acid positional distribution in colostrum and mature milk of women living in Inner Mongolia, North Jiangsu and Guangxi of China. Food & Function. 2018;9(8):4234-45.

55. Denic M, Sunaric S, Gencic M, Zivkovic J, Jovanovic T, Kocic G, et al. Maternal age has more pronounced effect on breast milk retinol and beta-carotene content than maternal dietary pattern. Nutrition. 2019;65:120-5.

56. Desantiago S, Ramirez I, Tovar AR, Ortiz N, Torres N, Bourges YH. Amino acid profiles in diet, plasma and human milk in Mexican rural lactating women. Nutrition Research. 1999;19(8):1133-43.

57. Ding M, Li W, Zhang Y, Wang X, Zhao A, Zhao X, et al. Amino acid composition of lactating mothers' milk and confinement diet in rural North China. Asia Pac J Clin Nutr. 2010;19(3):344-9.

58. Ding Y, Yang Y, Xu F, Ye M, Hu P, Jiang W, et al. Association between dietary fatty acid patterns based on principal component analysis and fatty acid compositions of serum and breast milk in lactating mothers in Nanjing, China. Food & Function. 2021;12(18):8704-14.

59. Duda G, Nogala-Kalucka M, Karwowska W, Kupczyk B, Lampart-Szczapa E. Influence of the lactating women diet on the concentration of the lipophilic vitamins in human milk. Pakistan Journal of Nutrition. 2009;8(5):629-34.

60. Dujmovic M, Kresic G, Mandic ML, Kenjeric D, Cvijanovic O. Changes in dietary intake and body weight in lactating and non-lactating women: Prospective study in northern coastal Croatia. Collegium Antropologicum. 2014;38(1):179-87.

61. Dumrongwongsiri O, Chongviriyaphan N, Chatvutinun S, Phoonlabdacha P, Sangcakul A, Siripinyanond A, et al. Dietary Intake and Milk Micronutrient Levels in Lactating Women with Full and Partial Breastfeeding. Maternal & Child Health Journal. 2021;25(6):991-7.

62. Elliott SA, Pereira LCR, McCargar LJ, Prado CM, Bell RC. Trajectory and determinants of change in lean soft tissue over the postpartum period. Br J Nutr. 2019;121(10):1137-45.

63. Filatava EJ, Shelly CE, Overton NE, Gregas M, Glynn R, Gregory KE. Human milk pH is associated with fortification, postpartum day, and maternal dietary intake in preterm mother-infant dyads. Journal of Perinatology. 2022.

64. Fiorella KJ, Milner EM, Bukusi E, Fernald LC. Quantity and species of fish consumed shape breast-milk fatty acid concentrations around Lake Victoria, Kenya. Public Health Nutr. 2018;21(4):777-84.

65. Fischer LM, da Costa KA, Galanko J, Sha W, Stephenson B, Vick J, et al. Choline intake and genetic polymorphisms influence choline metabolite concentrations in human breast milk and plasma. Am J Clin Nutr. 2010;92(2):336-46.

66. Fowles ER, Walker LO. Correlates of dietary quality and weight retention in postpartum women. Journal of Community Health Nursing. 2006;23(3):183-97.

67. Fujita M, Brindle E, Lo YJ, Castro P, Cameroamortegui F. Nutrient intakes associated with elevated serum C-reactive protein concentrations in normal to underweight breastfeeding women in Northern Kenya. American journal of human biology : the official journal of the Human Biology Council. 2014;26(6):796-802.

68. Fukushima Y, Kawata Y, Onda T, Kitagawa M. Comsumption of cow milk and egg by lactating women and the presence of beta-lactoglobulin and ovalbumin in breast milk. Am J Clin Nutr. 1997;65(1):30-5.

69. George AD, Gay MCL, Wlodek ME, Murray K, Geddes DT. The fatty acid species and quantity consumed by the breastfed infant are important for growth and development. Nutrients. 2021;13(11) (no pagination).

70. Gila-Diaz A, Diaz-Rullo Alcantara N, Herranz Carrillo G, Singh P, Arribas SM, Ramiro-Cortijo D. Multidimensional Approach to Assess Nutrition and Lifestyle in Breastfeeding Women during the First Month of Lactation. Nutrients. 2021;13(6):22.

71. Glew RH, Wold RS, Corl B, Calvin CD, Vanderjagt DJ. Low Docosahexaenoic Acid in the Diet and Milk of American Indian Women in New Mexico. Journal of the American Dietetic Association. 2011;111(5):744-8.

72. Glew RH, Wold RS, Herbein JH, Wark WA, Martinez MA, Vanderjagt DJ. Low docosahexaenoic acid in the diet and milk of women in New Mexico. Journal of the American Dietetic Association. 2008;108(10):1693-9.

73. Goetz LG, Valeggia C. The ecology of anemia: Anemia prevalence and correlated factors in adult indigenous women in Argentina. American journal of human biology : the official journal of the Human Biology Council. 2017;29(3).

74. Griebel-Thompson AK, Murray A, Morris KS, Paluch RA, Jacobson L, Kong KL. The Association between Maternal Sugar-Sweetened Beverage Consumption and Infant/Toddler Added Sugar Intakes. Nutrients. 2022;14(20) (no pagination).

75. Gupta C, Khedkar R, Negi K, Singh K. Undernutrition and associated factors among lactating mothers in Dehradun, Uttarakhand, India. Food Research. 2022;6(5):477-84.

76. Haire-Joshu D, Schwarz C, Budd E, Yount BW, Lapka C. Postpartum Teens' Breakfast Consumption Is Associated with Snack and Beverage Intake and Body Mass Index. Journal of the American Dietetic Association. 2011;111(1):124-30.

77. Hambidge KM, Miller LV, Mazariegos M, Westcott J, Solomons NW, Raboy V, et al. Upregulation of zinc absorption matches increases in physiologic requirements for zinc in women consuming high- or moderate-phytate diets during late pregnancy and early lactation. J Nutr. 2017;147(6):1079-85.

78. Han F, Pang X, Wang Q, Liu Y, Liu L, Chai Y, et al. Dietary Serine and Sulfate-Containing Amino Acids Related to the Nutritional Status of Selenium in Lactating Chinese Women. Biological Trace Element Research. 2021;199(3):829-41.

79. Hannan MA, Faraji B, Tanguma J, Longoria N, Rodriguez RC. Maternal milk concentration of zinc, iron, selenium, and iodine and its relationship to dietary intakes. Biological Trace Element Research. 2009;127(1):6-15.

80. Hasan M, Islam MM, Mubarak E, Haque MA, Choudhury N, Ahmed T. Mother's dietary diversity and association with stunting among children <2 years old in a low socio-economic environment: A case-control study in an urban care setting in Dhaka, Bangladesh. Matern Child Nutr. 2019;15(2) (no pagination).

81. Hattevig G, Kjellman B, Sigurs N, Grodzinsky E, Hed J, Bjorksten B. The effect of maternal avoidance of eggs, cow's milk, and fish during lactation on the development of IgE, IgG, and IgA antibodies in infants. Journal of Allergy and Clinical Immunology. 1990;85(1 I):108-15.

82. Hayat L, Al-Sughayer MA, Afzal M. Fatty acid composition of human milk in Kuwaiti mothers. Comparative Biochemistry and Physiology - B Biochemistry and Molecular Biology. 1999;124(3):261-7.

83. Heck H, De Castro JM. The caloric demand of lactation does not alter spontaneous meal patterns, nutrient intakes, or moods of women. Physiology and Behavior. 1993;54(4):641-8.

84. Henjum S, Lilleengen AM, Aakre I, Dudareva A, Gjengedal ELF, Meltzer HM, et al. Suboptimal Iodine Concentration in Breastmilk and Inadequate Iodine Intake among Lactating Women in Norway. Nutrients. 2017;9(7):22.

85. Henjum S, Manger M, Hampel D, Brantsaeter AL, Shahab-Ferdows S, Bastani NE, et al. Vitamin B12 concentrations in milk from Norwegian women during the six first months of lactation. Eur J Clin Nutr. 2020;74(5):749-56.

86. Henjum S, Manger M, Skeie E, Ulak M, Thorne-Lyman AL, Chandyo R, et al. Iron deficiency is uncommon among lactating women in urban Nepal, despite a high risk of inadequate dietary iron intake. Br J Nutr. 2014;112(1):132-41.

87. Hoppu U, Kalliomaki M, Isolauri E. Maternal diet rich in saturated fat during breastfeeding is associated with atopic sensitization of the infant. Eur J Clin Nutr. 2000;54(9):702-5.

88. Hoppu U, Rinne M, Salo-Vaananen P, Lampi AM, Piironen V, Isolauri E. Vitamin C in breast milk may reduce the risk of atopy in the infant. Eur J Clin Nutr. 2005;59(1):123-8.

89. Hoshino A, Yamada A, Tanabe R, Noda S, Nakaoka K, Oku Y, et al. Relationships between bone mass and dietary/lifestyle habits in Japanese women at 3-4 months postpartum. Public Health. 2017;152:129-35.

90. Howard K, Maples JM, Tinius RA. Modifiable Maternal Factors and Their Relationship to Postpartum Depression. Int J Environ Res Public Health. 2022;19(19) (no pagination).

91. Huang Q, Murphy J, Smith ER, Sylvetsky AC. Diet beverage intake during lactation and associations with infant outcomes in the infant feeding practices study II. Nutrients. 2021;13(9) (no pagination).

92. Huang Z, Hu YM. Dietary patterns and their association with breast milk macronutrient composition among lactating women. International Breastfeeding Journal. 2020;15(1):52.

93. Huseinovic E, Winkvist A, Bertz F, Bertéus Forslund H, Brekke HK. Eating frequency, energy intake and body weight during a successful weight loss trial in overweight and obese postpartum women. Eur J Clin Nutr. 2014;68(1):71-6.

94. Indriasari R, Syam A, Kurniati Y, Mansur MA. Zinc deficiency and inadequate zinc intake among postpartum women in coastal area of makassar, indonesia. Journal of Nutritional Science and Vitaminology. 2020;66(Supplement):S99-S102.

95. Jagodic M, Potocnik D, Snoj Tratnik J, Mazej D, Pavlin M, Trdin A, et al. Selected elements and fatty acid composition in human milk as indicators of seafood dietary habits. Environmental Research. 2020;180 (no pagination).

96. Jans G, Devlieger R, De Preter V, Ameye L, Roelens K, Lannoo M, et al. Bariatric Surgery Does Not Appear to Affect Women's Breast-Milk Composition. The Journal of nutrition. 2018;148(7):1096-102.

97. Jans G, Devlieger R, Preter VD, Ameye L, Roelens K, Lannoo M, et al. Bariatric Surgery Does Not Appear to Affect Women's Breast-Milk Composition. J Nutr. 2018;148(7):1096-102.

98. Jans G, Matthys C, Lannoo M, Van der Schueren B, Devlieger R. Breast milk macronutrient composition after bariatric surgery. Obesity Surgery. 2015;25(5):938-41.

99. Jiang J, Wu K, Yu Z, Ren Y, Zhao Y, Jiang Y, et al. Changes in fatty acid composition of human milk over lactation stages and relationship with dietary intake in Chinese women. Food & Function. 2016;7(7):3154-62.

100. Jiang J, Xiao H, Wu K, Yu Z, Ren Y, Zhao Y, et al. Retinol and alpha-tocopherol in human milk and their relationship with dietary intake during lactation. Food & Function. 2016;7(4):1985-91.

101. Jiang W, Mo M, Li M, Wang S, Muyiduli X, Shao B, et al. The relationship of dietary diversity score with depression and anxiety among prenatal and post-partum women. J Obstet Gynaecol Res. 2018;44(10):1929-36.

102. Jin Y, Coad J, Pond R, Kim N, Brough L. Selenium intake and status of postpartum women and postnatal depression during the first year after childbirth in New Zealand - Mother and Infant Nutrition Investigation (MINI) study. Journal of Trace Elements in Medicine and Biology. 2020;61 (no pagination).

103. Jin Y, Coad J, Weber JL, Thomson JS, Brough L. Selenium Intake in Iodine-Deficient Pregnant and Breastfeeding Women in New Zealand. Nutrients. 2019;11(1):01.

104. Jirapinyo P, Densupsoontorn N, Wiraboonchai D, Vissavavejam U, Tangtrakulvachira T, Chungsomprasong P, et al. Fatty acid composition in breast milk from 4 regions of Thailand. Journal of the Medical Association of Thailand. 2008;91(12):1833-7.

105. Johnston L, Vaughan L, Fox HM. Pantothenic acid content of human milk. Am J Clin Nutr. 1981;34(10):2205-9.

106. Jonsson K, Barman M, Moberg S, Sjoberg A, Brekke HK, Hesselmar B, et al. Fat intake and breast milk fatty acid composition in farming and nonfarming women and allergy development in the offspring. Pediatric Research. 2016;79(1):114-23.

107. Kaliwile C, Michelo C, Sheftel J, Davis CR, Grahn M, Bwembya P, et al. Breast Milk-Derived Retinol Is a Potential Surrogate for Serum in the <sup>13</sup>C-Retinol Isotope Dilution Test in Zambian Lactating Women with Vitamin A Deficient and Adequate Status. J Nutr. 2021;151(1):255-63.

108. Karbasi S, Bahrami A, Asadi Z, Shahbeiki F, Naseri M, Zarban A, et al. The association of maternal dietary quality and the antioxidant-proxidant balance of human milk. International Breastfeeding Journal. 2022;17(1):1-9.

109. Kay MC, Wasser H, Adair LS, Thompson AL, Siega-Riz AM, Suchindran CM, et al. Consumption of obesogenic foods in non-Hispanic black mother-infant dyads. Matern Child Nutr. 2018;14(1) (no pagination).

110. Keller BO, Wu BT, Li SS, Monga V, Innis SM. Hypaphorine is present in human milk in association with consumption of legumes. Journal of Agricultural & Food Chemistry. 2013;61(31):7654-60.

111. Kesa H, Oldewage-Theron W. Anthropometric indications and nutritional intake of women in the Vaal Triangle, South Africa. Public Health. 2005;119(4):294-300.

112. Khymenets O, Rabassa M, Rodriguez-Palmero M, Rivero-Urgell M, Urpi-Sarda M, Tulipani S, et al. Dietary Epicatechin Is Available to Breastfed Infants through Human Breast Milk in the Form of Host and Microbial Metabolites. Journal of agricultural and food chemistry. 2016;64(26):5354-60.

113. Kim H, Kang S, Jung BM, Yi H, Jung JA, Chang N. Breast milk fatty acid composition and fatty acid intake of lactating mothers in South Korea. Br J Nutr. 2017;117(4):556-61.

114. Kim H, Yi H, Jung JA, Chang N. Association between lutein intake and lutein concentrations in human milk samples from lactating mothers in South Korea. Eur J Nutr. 2018;57(1):417-21.

115. Krebs NF, Reidinger CJ, Robertson AD, Brenner M. Bone mineral density changes during lactation: maternal, dietary, and biochemical correlates. Am J Clin Nutr. 1997;65(6):1738-46.

116. Laskey MA, Prentice A, Hanratty LA, Jarjou LMA, Dibba B, Beavan SR, et al. Bone changes after 3 mo of lactation: influence of calcium intake, breast-milk output, and vitamin D-receptor genotype... including commentary by Allen LH. Am J Clin Nutr. 1998;67(4):685-592.

117. Lee DK, Lee H, Yoon T, Park SJ, Lee HJ. Nationwide representative survey of dietary iodine intake and urinary excretion in postpartum korean women. Nutrients. 2021;13(11) (no pagination).

118. Leelahakul V, Tanaka F, Sinsuksai N, Vichitsukon K, Pinyopasakul W, Kido N, et al. Comparison of the protein composition of breast milk and the nutrient intake between Thai and Japanese mothers. Nursing and Health Sciences. 2009;11(2):180-4.

119. Lemay-Nedjelski L, Asbury MR, Butcher J, Ley SH, Hanley AJ, Kiss A, et al. Maternal Diet and Infant Feeding Practices Are Associated with Variation in the Human Milk Microbiota at 3 Months Postpartum in a Cohort of Women with High Rates of Gestational Glucose Intolerance. J Nutr. 2021;151(2):320-9.

120. Leung CW, Laraia BA, Coleman-Phox K, Bush NR, Lin J, Blackburn EH, et al. Sugary beverage and food consumption, and leukocyte telomere length maintenance in pregnant women. Eur J Clin Nutr. 2016;70(9):1086-8.

121. Li N, Su X, Liu T, Sun J, Zhu Y, Dai Z, et al. Dietary patterns of Chinese puerperal women and their association with postpartum weight retention: Results from the mother-infant cohort study. Matern Child Nutr. 2021;17(1) (no pagination).

122. Lioret S, Cameron AJ, McNaughton SA, Crawford D, Spence AC, Hesketh K, et al. Association between maternal education and diet of children at 9 months is partially explained by mothers' diet. Matern Child Nutr. 2015;11(4):936-47.

123. Little RE, Northstone K, Golding J. Alcohol, breastfeeding, and development at 18 months. Pediatrics. 2002;109(5):6p-p.

124. Liu G, Ding Z, Li X, Chen X, Wu Y, Xie L. Relationship between polyunsaturated fatty acid levels in maternal diets and human milk in the first month post-partum. Journal of Human Nutrition & Dietetics. 2016;29(4):405-10.

125. Liu L, Guo Q, Cui M, Liu J, Yang C, Li X, et al. Impact of maternal nutrition during early pregnancy and diet during lactation on lactoferrin in mature breast milk. Nutrition. 2022;93 (no pagination).

126. Liu MJ, Li HT, Yu LX, Xu GS, Ge H, Wang LL, et al. A correlation study of DHA dietary intake and plasma, erythrocyte and breast milk DHA concentrations in lactatingwomen from Coastland, Lakeland, and Inland areas of China. Nutrients. 2016;8(5) (no pagination).

127. Lu M, Jiang J, Wu K, Li D. Epidermal growth factor and transforming growth factor-alpha in human milk of different lactation stages and different regions and their relationship with maternal diet. Food & Function. 2018;9(2):1199-204.

128. Lu M, Xiao H, Li K, Jiang J, Wu K, Li D. Concentrations of estrogen and progesterone in breast milk and their relationship with the mother's diet. Food & Function. 2017;8(9):3306-10.

129. Lu Z, Chan YT, Lo KK, Wong VW, Ng YF, Li SY, et al. Levels of polyphenols and phenolic metabolites in breast milk and their association with plant-based food intake in Hong Kong lactating women. Food & Function. 2021;12(24):12683-95.

130. Lu Z, Chan YT, Lo KKH, Wong VWS, Ng YF, Li SY, et al. Levels of polyphenols and phenolic metabolites in breast milk and their association with plant-based food intake in Hong Kong lactating women. Food & Function. 2021;12(24):12683-95.

131. Lu Z, Chan YT, Lo KKH, Zhao D, Wong VWS, Ng YF, et al. Carotenoids and Vitamin A in Breastmilk of Hong Kong Lactating Mothers and Their Relationships with Maternal Diet. Nutrients. 2022;14(10) (no pagination).

132. Lumia M, Luukkainen P, Kaila M, Tapanainen H, Takkinen HM, Prasad M, et al. Maternal dietary fat and fatty acid intake during lactation and the risk of asthma in the offspring. Acta Paediatrica, International Journal of Paediatrics. 2012;101(8):e337-e43.

133. Lundqvist A, Sandstrom H, Stenlund H, Johansson I, Hultdin J. Vitamin D status during pregnancy: A longitudinal study in Swedish women from early pregnancy to seven months postpartum. PLoS ONE [Electronic Resource]. 2016;11(3) (no pagination).

134. Lyu LC, Lo CC, Chen HF, Wang CY, Liu DM. A prospective study of dietary intakes and influential factors from pregnancy to postpartum on maternal weight retention in Taipei, Taiwan. Br J Nutr. 2009;102(12):1828-37.

135. Machado MR, Kamp F, Nunes JC, El-Bacha T, Torres AG. Breast Milk Content of Vitamin A and E from Early- to Mid-Lactation Is Affected by Inadequate Dietary Intake in Brazilian Adult Women. Nutrients. 2019;11(9):2025.

136. Mahdavi R, Nikniaz L, Arefhosseini SR, Jabbari MV. Determination of aflatoxin M1 in breast milk samples in Tabriz-Iran. Maternal & Child Health Journal. 2010;14(1):141-5.

137. Mao Y, Liu S, Wang J, Li X, Zhao Y, Hill DR, et al. Vitamins, Vegetables and Metal Elements Are Positively Associated with Breast Milk Oligosaccharide Composition among Mothers in Tianjin, China. Nutrients. 2022;14(19) (no pagination).

138. Martysiak-Zurowska D, Zagierski M, Wos-Wasilewska E, Szlagatys-Sidorkiewicz A. Higher absorption of vitamin C from food than from supplements by breastfeeding mothers at early stages of lactation. International Journal for Vitamin and Nutrition Research. 2016;86(3-4):81-7.

139. Mereu R, Atzori L, Mereu A, Galassi S, Corda S, Frongia P, et al. High meat consumption is associated with type 1 diabetes mellitus in a Sardinian case-control study. Acta Diabetologica. 2013;50(5):713-9.

140. Miliku K, Duan QL, Moraes TJ, Becker AB, Mandhane PJ, Turvey SE, et al. Human milk fatty acid composition is associated with dietary, genetic, sociodemographic, and environmental factors in the CHILD Cohort Study. Am J Clin Nutr. 2019;110(6):1370-83.

141. Minato T, Nomura K, Asakura H, Aihara A, Hiraike H, Hino Y, et al. Maternal undernutrition and breast milk macronutrient content are not associated with weight in breastfed infants at 1 and 3 months after delivery. Int J Environ Res Public Health. 2019;16(18) (no pagination).

142. Miranda AR, Cortez MV, Scotta AV, Rivadero L, Serra SV, Soria EA. Memory enhancement in Argentinian women during postpartum by the dietary intake of lignans and anthocyanins. Nutrition Research. 2021;85:1-13.

143. Mitchell ME, Snyder EA. Dietary carnitine effects on carnitine concentrations in urine and milk in lactating women. Am J Clin Nutr. 1991;54(5):814-20.

144. Mojska H, Socha P, Socha J, Soplinska E, Jaroszewska-Balicka W, Szponar L. Trans fatty acids in human milk in Poland and their association with breastfeeding mothers' diets. Acta Paediatrica, International Journal of Paediatrics. 2003;92(12):1381-7.

145. Moltó-Puigmartí C, Plat J, Mensink RP, Müller A, Jansen E, Zeegers MP, et al. FADS1 FADS2 gene variants modify the association between fish intake and the docosahexaenoic acid proportions in human milk. Am J Clin Nutr. 2010;91(5):1368-76.

146. Montez de Sousa i R, Wang Z, Hu R, Stahl B, Jin Y, Eussen SR, et al. Dietary Intake of Chinese Lactating Women Is Associated with the Fatty Acid Profile of Their Milk. Annals of Nutrition and Metabolism. 2022;78(1):33-45.

147. Moon S, Kim J. Iodine content of human milk and dietary iodine intake of Korean lactating mothers. International Journal of Food Sciences and Nutrition. 1999;50(3):165-71.

148. Morton SU, Vyas R, Gagoski B, Vu C, Litt J, Larsen RJ, et al. Maternal Dietary Intake of Omega-3 Fatty Acids Correlates Positively with Regional Brain Volumes in 1-Month-Old Term Infants. Cerebral Cortex. 2020;30(4):2057-69.

149. Moser PB, Issa CF, Reynolds RD. Dietary magnesium intake and the concentration of magnesium in plasma and erythrocytes of postpartum women. Journal of the American College of Nutrition. 1983;2(4):387-96.

150. Motil KJ, Sheng H, Kertz BL, Montandon CM, Ellis KJ. Lean body mass of well-nourished women is preserved during lactation. Am J Clin Nutr. 1998;67(2):292-300.

151. Motoyama K, Isojima T, Sato Y, Aihara A, Asakura H, Hiraike H, et al. Trace element levels in mature breast milk of recently lactating Japanese women. Pediatrics International. 2021;63(8):910-7.

152. Nagayasu Y, Fujita D, Daimon A, Nunode M, Sawada M, Sano T, et al. Possible prevention of post-partum depression by intake of omega-3 polyunsaturated fatty acids and its relationship with interleukin 6. J Obstet Gynaecol Res. 2021;47(4):1371-9.

153. Nagel EM, Jacobs D, Johnson KE, Foster L, Duncan K, Kharbanda EO, et al. Maternal Dietary Intake of Total Fat, Saturated Fat, and Added Sugar Is Associated with Infant Adiposity and Weight Status at 6 mo of Age. J Nutr. 2021;151(8):2353-60.

154. Nakai S, Tateoka Y, Miyaguchi Y, Takahashi M, Ogita H. Associations Between Habitual Dietary Behaviors and Glutamic Acid Levels in Human Milk. Journal of human lactation : official journal of International Lactation Consultant Association. 2022:8903344221095784.

155. Neumann CG, Oace SM, Chaparro MP, Herman D, Drorbaugh N, Bwibo NO. Low vitamin B12 intake during pregnancy and lactation and low breastmilk vitamin 12 content in rural Kenyan women consuming predominantly maize diets. Food Nutr Bull. 2013;34(2):151-9.

156. Nicklas JM, Zera CA, Seely EW. Predictors of very early postpartum weight loss in women with recent gestational diabetes mellitus. Journal of Maternal-Fetal and Neonatal Medicine. 2020;33(1):120-6.

157. Niinisto S, Takkinen HM, Uusitalo L, Rautanen J, Vainio N, Ahonen S, et al. Maternal intake of fatty acids and their food sources during lactation and the risk of preclinical and clinical type 1 diabetes in the offspring. Acta Diabetologica. 2015;52(4):763-72.

158. Nikniaz L, Mahdavi R, Arefhoesseini S, Khiabani MS. Association between fat content of breast milk and maternal nutritional status and infants' weight in Tabriz, Iran. Malays J Nutr. 2009;15(1):37-44.

159. Nimmannun K, Davis CR, Srisakda P, Gannon BM, Tanumihardjo SA, Udomkesmalee E. Breast Milk Retinol Concentrations Reflect Total Liver Vitamin a Reserves and Dietary Exposure in Thai Lactating Women from Urban and Rural Areas. The Journal of nutrition. 2022;28.

160. Nishimura RY, Barbieiri P, de Castro GSF, Jordao AA, da Silva Castro Perdona G, Sartorelli DS. Dietary polyunsaturated fatty acid intake during late pregnancy affects fatty acid composition of mature breast milk. Nutrition. 2014;30(6):685-9.

161. Niwa S, Kawabata T, Shoji K, Ogata H, Kagawa Y, Nakayama K, et al. Investigation of Maternal Diet and FADS1 Polymorphism Associated with Long-Chain Polyunsaturated Fatty Acid Compositions in Human Milk. Nutrients. 2022;14(10) (no pagination).

162. Nommsen LA, Lovelady CA, Heinig MJ, Lonnerdal B, Dewey KG. Determinants of energy, protein, lipid, and lactose concentrations in human milk during the first 12 mo of lactation: The DARLING study. Am J Clin Nutr. 1991;53(2):457-65.

163. O'Brien KO, Donangelo CM, Zapata CLV, Abrams SA, Spencer EM, King JC. Bone calcium turnover during pregnancy and lactation in women with low calcium diets is associated with calcium intake and circulating insulin-like growth factor 1 concentrations. Am J Clin Nutr. 2006;83(2):317-23.

164. Oken E, Taveras EM, Popoola FA, Rich-Edwards JW, Gillman MW. Television, Walking, and Diet. Associations with Postpartum Weight Retention. American Journal of Preventive Medicine. 2007;32(4):305-11.

165. Olafsdottir AS, Thorsdottir I, Wagner KH, Elmadfa I. Polyunsaturated fatty acids in the diet and breast milk of lactating Icelandic women with traditional fish and cod liver oil consumption. Annals of Nutrition and Metabolism. 2006;50(3):270-6.

166. Olafsdottir AS, Wagner KH, Thorsdottir I, Elmadfa I. Fat-soluble vitamins in the maternal diet, influence of cod liver oil supplementation and impact of the maternal diet on human milk composition. Annals of Nutrition & Metabolism. 2001;45(6):265-72.

167. Olson CM, Strawderman MS, Hinton PS, Pearson TA. Gestational weight gain and postpartum behaviors associated with weight change from early pregnancy to 1 y postpartum. International Journal of Obesity. 2003;27(1):117-27.

168. Otto SJ, Van Houwelingen AC, Badart-Smook A, Hornstra G. Comparison of the peripartum and postpartum phospholipid polyunsaturated fatty acid profiles of lactating and nonlactating women. Am J Clin Nutr. 2001;73(6):1074-9.

169. Park Y, McGuire MK, Behr R, McGuire MA, Evans MA, Shultz TD. High-fat dairy product consumption increases delta 9c,11t-18:2 (rumenic acid) and total lipid concentrations of human milk. Lipids. 1999;34(6):543-9.

170. Pauwels S, Ghosh M, Duca RC, Bekaert B, Freson K, Huybrechts I, et al. Maternal intake of methyl-group donors affects DNA methylation of metabolic genes in infants. Clinical Epigenetics. 2017;9(1) (no pagination).

171. Purkiewicz A, Pietrzak-Fiecko R, Sorgel F, Kinzig M. Caffeine, Paraxanthine, Theophylline, and Theobromine Content in Human Milk. Nutrients. 2022;14(11) (no pagination).

172. Qiao Y, Feng J, Yang J, Gu G. The relationship between dietary vitamin A intake and the levels of sialic acid in the breast milk of lactating women. Journal of Nutritional Science & Vitaminology. 2013;59(4):347-51.

173. Qin Y, Chen Y, Huang S, Jiao C, Zhang Z, Mao L. Associations of dietary inflammatory potential with postpartum weight change and retention: Results from a cohort study. Obesity. 2021;29(10):1689-99.

174. Qin Y, Jiao C, Huang S, Li Y, Zhang Z, Bao W, et al. "Zuoyuezi" dietary and behavioural associations with maternal health among puerperal women in South China. Asia Pac J Clin Nutr. 2021;30(2):291-302.

175. Quansah DY, Schenk S, Gilbert L, Arhab A, Gross J, Marques-Vidal PM, et al. Intuitive Eating Behavior, Diet Quality and Metabolic Health in the Postpartum in Women with Gestational Diabetes. Nutrients. 2022;14(20) (no pagination).

176. Quinn E, Kuzawa C. A dose-response relationship between fish consumption and human milk DHA content among Filipino women in Cebu City, Philippines. Acta Paediatr. 2012;101(10):e439-45.

177. Quinn EA, Kuzawa CW. A dose-response relationship between fish consumption and human milk DHA content among Filipino women in Cebu City, Philippines. Acta Paediatrica, International Journal of Paediatrics. 2012;101(10):e439-e45.

178. Quinn EA, Largado F, Power M, Kuzawa CW. Predictors of breast milk macronutrient composition in filipino mothers. Am J Hum Biol. 2012;24(4):533-40.

179. Rosinger AY, Lawman HG, Akinbami LJ, Ogden CL. The role of obesity in the relation between total water intake and urine osmolality in US adults, 2009-20121-3. Am J Clin Nutr. 2016;104(6):1554-61.

180. Roy S, Dhar P, Ghosh S, Roy S, Dhar P, Ghosh S. Comparative evaluation of essential fatty acid composition of mothers' milk of some urban and suburban regions of West Bengal, India. International Journal of Food Sciences & Nutrition. 2012;63(8):895-901.

181. Samur G, Topcu A, Turan S. Trans fatty acids and fatty acid composition of mature breast milk in turkish women and their association with maternal diet's. Lipids. 2009;44(5):405-13.

182. Sandjaja, Jus’at I, Jahari AB, Ifrad, Htet MK, Tilden RL, et al. Vitamin A-fortified cooking oil reduces vitamin A deficiency in infants, young children and women: results from a programme evaluation in Indonesia. Public Health Nutr. 2015;18(14):2511-22.

183. Scopesi F, Ciangherotti S, Lantieri PB, Risso D, Bertini I, Campone F, et al. Maternal dietary PUFAs intake and human milk content relationships during the first month of lactation. Clinical Nutrition. 2001;20(5):393-7.

184. Sekine Y, Matsunaga N, Kokaze A, Yoshida M, Suzuki K, Ohno H, et al. Effects of nutrient and food intake on calcaneous bone mass among healthy Japanese women in the predelivery and postpartum periods. Journal of Women's Health (15409996). 2003;12(7):643-54.

185. Shah BS, Freeland-Graves JH, Cahill JM, Lu H, Graves GR. Diet Quality as Measured by the Healthy Eating Index and the Association with Lipid Profile in Low-Income Women in Early Postpartum. Journal of the American Dietetic Association. 2010;110(2):274-9.

186. Shen Y, Huang L, Zou Y, Su D, He M, Fang Y, et al. Intake of Vitamin B12 and Folate and Biomarkers of Nutrient Status of Women within Two Years Postpartum. Nutrients. 2022;14(18) (no pagination).

187. Sian L, Krebs NF, Westcott JE, Fengliang L, Tong L, Miller LV, et al. Zinc homeostasis during lactation in a population with a low zinc intake. Am J Clin Nutr. 2002;75(1):99-103.

188. Sindi AS, Stinson LF, Lean SS, Chooi YH, Leghi GE, Netting MJ, et al. Effect of a reduced fat and sugar maternal dietary intervention during lactation on the infant gut microbiome. Frontiers in Microbiology. 2022;13 (no pagination).

189. Specker BL, Tsang RC, Hollis BW. Effect of race and diet on human-milk vitamin D and 25-hydroxyvitamin D. American Journal of Diseases of Children. 1985;139(11):1134-7.

190. Stendell-Hollis NR, Thompson PA, West JL, Wertheim BC, Thomson CA. A Comparison of Mediterranean-Style and MyPyramid Diets on Weight Loss and Inflammatory Biomarkers in Postpartum Breastfeeding Women. Journal of Women's Health (15409996). 2013;22(1):48-57.

191. Stravik M, Barman M, Hesselmar B, Sandin A, Wold AE, Sandberg AS. Maternal intake of cow's milk during lactation is associated with lower prevalence of food allergy in offspring. Nutrients. 2020;12(12):1-19.

192. Su X, Zhu W, Li N, Sun J, Zhu Y, Liu T, et al. Adjusting DBI-2016 to dietary balance index for Chinese maternal women and assessing the association between maternal dietary quality and postpartum weight retention: A longitudinal study. PLoS ONE [Electronic Resource]. 2020;15(8 August) (no pagination).

193. Tahir MJ, Haapala JL, Foster LP, Duncan KM, Teague AM, Kharbanda EO, et al. Higher maternal diet quality during pregnancy and lactation is associated with lower infant weight-for-length, body fat percent, and fat mass in early postnatal life. Nutrients. 2019;11(3) (no pagination).

194. Tang N, Wu Y, Chen Y, Chen Q, Wu W, Jing J, et al. Association between postpartum low-carbohydrate-diet scores and glucose levels in Chinese women. Nutrition. 2021;89 (no pagination).

195. Tawfeek HI, Muhyaddin OM, Al-Sanwi HI, Al-Baety N. Effect of maternal dietary vitamin C intake on the level of vitamin C in breastmilk among nursing mothers in Baghdad, Iraq. Food Nutr Bull. 2002;23(3):244-7.

196. Teo C, Chia AR, Colega MT, Chen LW, Fok D, Pang WW, et al. Prospective Associations of Maternal Dietary Patterns and Postpartum Mental Health in a Multi-Ethnic Asian Cohort: The Growing up in Singapore towards Healthy Outcomes (GUSTO) Study. Nutrients. 2018;10(3):02.

197. Thakur A, Kler N, Garg P, Gandhi P, Srivastava S. Macronutrient analysis of human milk and factors associated with its composition in mothers of preterm infants <= 32 weeks. Eur J Pediatr. 2021;180(12):3527-34.

198. Tian HM, Wu YX, Lin YQ, Chen XY, Yu M, Lu T, et al. Dietary patterns affect maternal macronutrient intake levels and the fatty acid profile of breast milk in lactating Chinese mothers. Nutrition. 2019;58:83-8.

199. Tikuye HH, Gebremedhin S, Mesfin A, Whiting S. Prevalence and Factors Associated with Undernutrition among Exclusively Breastfeeding Women in Arba Minch Zuria District, Southern Ethiopia: A Cross-sectional Community-Based Study. Ethiop J Health Sci. 2019;29(1):913-22.

200. Tuokkola J, Luukkainen P, Kaila M, Takkinen HM, Niinisto S, Veijola R, et al. Maternal dietary folate, folic acid and Vitamin D intakes during pregnancy and lactation and the risk of cows' milk allergy in the offspring. Br J Nutr. 2016;116(4):710-8.

201. Tuokkola J, Luukkainen P, Tapanainen H, Kaila M, Vaarala O, Kenward MG, et al. Maternal diet during pregnancy and lactation and cow's milk allergy in offspring. Eur J Clin Nutr. 2016;70(5):554-9.

202. Ueno HM, Higurashi S, Shimomura Y, Wakui R, Matsuura H, Shiota M, et al. Association of DHA Concentration in Human Breast Milk with Maternal Diet and Use of Supplements: A Cross-Sectional Analysis of Data from the Japanese Human Milk Study Cohort. Curr Dev Nutr. 2020;4(7) (no pagination).

203. Ueno HM, Sato T, Higurashi S, Tazaki H, Toba Y. Xanthophylls in Human Milk and Maternal Diet: A Cross-sectional Analysis of Data from the Japanese Human Milk Study Cohort. Curr Dev Nutr. 2022;6(6).

204. Ureta-Velasco N, Keller K, Escuder-Vieco D, Serrano JCE, Garcia-Lara NR, Pallas-Alonso CR. Assessment of Iodine Concentration in Human Milk from Donors: Implications for Preterm Infants. Nutrients. 2022;14(20) (no pagination).

205. Vähämiko S, Isolauri E, Laitinen K. Weight status and dietary intake determine serum leptin concentrations in pregnant and lactating women and their infants. Br J Nutr. 2013;110(6):1098-106.

206. Valent F, Horvat M, Mazej D, Stibilj V, Barbone F. Maternal diet and selenium concentration in human milk from an Italian population. Journal of Epidemiology. 2011;21(4):285-92.

207. Villalpando S, Latulippe ME, Rosas G, Irurita MJ, Picciano MF, O'Connor DL. Milk folate but not milk iron concentrations may be inadequate for some infants in a rural farming community in San Mateo, Capulhuac, Mexico. Am J Clin Nutr. 2003;78(4):782-9.

208. Ward MH, Pan WH, Cheng YJ, Li FH, Brinton LA, Chen CJ, et al. Dietary exposure to nitrite and nitrosamines and risk of nasopharyngeal carcinoma in Taiwan. Int J Cancer. 2000;86(5):603-9.

209. Williams AM, Chantry CJ, Young SL, Achando BS, Allen LH, Arnold BF, et al. Vitamin B-12 Concentrations in Breast Milk Are Low and Are Not Associated with Reported Household Hunger, Recent Animal-Source Food, or Vitamin B-12 Intake in Women in Rural Kenya. J Nutr. 2016;146(5):1125-31.

210. Williams JE, Carrothers JM, Lackey KA, Beatty NF, York MA, Brooker SL, et al. Human Milk Microbial Community Structure Is Relatively Stable and Related to Variations in Macronutrient and Micronutrient Intakes in Healthy Lactating Women. J Nutr. 2017;147(9):1739-48.

211. Wiltheiss GA, Lovelady CA, West DG, Brouwer RJ, Krause KM, Ostbye T. Diet quality and weight change among overweight and obese postpartum women enrolled in a behavioral intervention program. Journal of the Academy of Nutrition & Dietetics. 2013;113(1):54-62.

212. Wong VWS, Ng YF, Chan SM, Su YX, Kwok KWH, Chan HM, et al. Positive relationship between consumption of specific fish type and n-3 PUFA in milk of Hong Kong lactating mothers. Br J Nutr. 2019;121(12):1431-40.

213. Wu W-C, Lin H-C, Liao W-L, Tsai Y-Y, Chen A-C, Chen H-C, et al. FADS Genetic Variants in Taiwanese Modify Association of DHA Intake and Its Proportions in Human Milk. Nutrients. 2020;12(2):543.

214. Xiang M, Harbige L, Zetterstrom R. Long-chain polyunsaturated fatty acids in Chinese and Swedish mothers: Diet, breast milk and infant growth. Acta Paediatrica, International Journal of Paediatrics. 2005;94(11):1543-9.

215. Xiao R, Moore Simas T, Pagoto S, Person S, Rosal M, Waring M. Sleep Duration and Diet Quality Among Women Within 5 Years of Childbirth in the United States: A Cross-Sectional Study. Maternal & Child Health Journal. 2016;20(9):1869-77.

216. Xu X, Zhao X, Berde Y, Low YL, Kuchan MJ. Milk and Plasma Lutein and Zeaxanthin Concentrations in Chinese Breast-Feeding Mother-Infant Dyads With Healthy Maternal Fruit and Vegetable Intake. Journal of the American College of Nutrition. 2019;38(2):179-84.

217. Xue Y, Campos-Gimenez E, Redeuil KM, Leveques A, Actis-Goretta L, Vinyes-Pares G, et al. Concentrations of carotenoids and tocopherols in breast milk from urban chinese mothers and their associations with maternal characteristics: A cross-sectional study. Nutrients. 2017;9(11) (no pagination).

218. Xuto P, Sinsuksai N, Piaseu N, Nityasuddhi D, Phupong V. A Causal Model of Postpartum Weight Retention among Thais. Pacific Rim International Journal of Nursing Research. 2012;16(1):48-63.

219. Yang J, Zheng H, Li X, Zhu L, Hao Z, Chen G, et al. Assessment of iodine status and associated factors in vulnerable populations in Henan Province, China, in 2012. Asia Pac J Clin Nutr. 2014;23(4):626-33.

220. Yang T, Zhang Y, Ning Y, You L, Ma D, Zheng Y, et al. Breast milk macronutrient composition and the associated factors in urban Chinese mothers. Chinese Medical Journal. 2014;127(9):1721-5.

221. Yang Y, Li G, Li F, Xu F, Hu P, Xie Z, et al. Impact of DHA from Algal Oil on the Breast Milk DHA Levels of Lactating Women: A Randomized Controlled Trial in China. Nutrients. 2022;14(16) (no pagination).

222. Yilmaz B, Sandal S, Ayvaci H, Tug N, Vitrinel A. Genotoxicity profiles in exfoliated human mammary cells recovered from lactating mothers in Istanbul; relationship with demographic and dietary factors. Mutation Research - Genetic Toxicology and Environmental Mutagenesis. 2012;749(1-2):17-22.

223. Yu Y, Wang B, Wang X, Wang R, Wang W, Shen G, et al. Hexachlorocyclohexanes (HCHs) in placenta and umbilical cord blood and dietary intake for women in Beijing, China. Environmental Pollution. 2013;179:75-80.

224. Zhang Y, Gu W, Zhao X, Shan L, Zhang Z, Wu X, et al. Estimation of appropriate dietary intake of iodine among lactating women in China based on iodine loss in breast milk. European Journal of Nutrition. 2022.

225. Zhang Y, Yang J, Huang N, Xiao L, Lin H, Luo J, et al. Changes in breast milk lutein concentrations and their associations with dietary lutein intake: A 12-week prospective analytical study. Br J Nutr. 2019;122(9):1033-9.

226. Zhao A, Huo S, Tan Y, Yang Y, Szeto IMY, Zhang Y, et al. The Association between Postpartum Practice and Chinese Postpartum Depression: Identification of a Postpartum Depression-Related Dietary Pattern. Nutrients. 2022;14(4) (no pagination).

227. Zhao A, Ning YB, Zhang YM, Yang XG, Wang JK, Li WJ, et al. Mineral compositions in breast milk of healthy chinese lactating women in urban areas and its associated factors. Chinese Medical Journal. 2014;127(14):2643-8.

228. Zhou Y, Zhu X, Zhang M, Li Y, Liu W, Huang H, et al. Association between dietary inflammatory index and bone density in lactating women at 6months postpartum: a longitudinal study. BMC Public Health. 2019;19(1):1076.

229. Zielinska MA, Hamulka J, Wesolowska A. Carotenoid Content in Breastmilk in the 3rd and 6th Month of Lactation and Its Associations with Maternal Dietary Intake and Anthropometric Characteristics. Nutrients. 2019;11(1):193.

230. Zielinska-Pukos MA, Brys J, Wesolowska A, Hamulka J. Breastmilk PUFA strongly associated with maternal dietary intake but not anthropometric parameters and breastmilk carotenoids. Prostaglandins Leukotrienes and Essential Fatty Acids. 2022;186 (no pagination).

231. Zou H, Sun M, Liu Y, Xi Y, Xiang C, Yong C, et al. Relationship between Dietary Inflammatory Index and Postpartum Depression in Exclusively Breastfeeding Women. Nutrients. 2022;14(23) (no pagination).
